# Supplementary material for: Early DNA methylation at the NGFI-A binding site of the NR3C1 1F promoter predicts cognitive functions at age five: Evidence from the Parents as Teachers intervention in the ZEPPELIN study
Source: PLoS One. 2026 Jul 20;21(7):e0344401. doi: 10.1371/journal.pone.0344401 (PMC13384299; doi:10.1371/journal.pone.0344401)
Supplement: S1 Text — (DOCX) [file pone.0344401.s001.docx]

**S1 Text. Detailed R Code and Output for Statistical Analyses**

1. **Study variables and descriptive statistics**
2. **Handling of Missing Data**
3. **Regression Models (Models 1-4)**
4. **Mediation Models (Models 5-7)**
5. **Moderated and Serial Mediation Models (Models 8-10)**
6. **Sensitivity analyses (Complete cases & NMAR)**
7. **Study variables and descriptive statistics**

| **cngrup** | PAT Group |
| --- | --- |
| **cnsex** | Gender |
| **cnrandalt** | Age at T0 |
| **cngew** | Birth weight |
| **Europe_vs_Others** | Geographical Origin |
| **h3isei** | Socio-economic status (SES) |
| **logMeth** | DNA methylation |
| **h3tage** | Age at T3 |
| **son5iq** | IQ |
| **son5kon** | Concentration |
| **son5ver** | Understanding of the instructions |
| **son5mot** | Motivation |
| **son5koo** | Cooperation |
| **son5mon** | Age at T5 |
| **e3part** | Parental disagreement |
| **cnerr** | Family outreach difficulty |

**Analytical study sample vs full cohort comparison**

**Sex, SES, age at randomization**

p_sex <- chisq.test(table(dati_zeppelin$group, dati_zeppelin$cnsex))$p.value

> p_hisei <- wilcox.test(hisei ~ group, data = dati_zeppelin)$p.value

> p_rand <- wilcox.test(cnrandalt ~ group, data = dati_zeppelin)$p.value

> results <- data.frame(

+ Variable = c("cnsex", "hisei", "cnrandalt"),

+ Type = c("Categorical", "Continuous", "Continuous"),

+ Test = c("Chi-square", "Wilcoxon", "Wilcoxon"),

+ p_value = c(p_sex, p_hisei, p_rand)

+ )

> results$Interpretation <- ifelse(results$p_value < 0.05,

+ "SIGNIFICANT difference",

+ "NOT significant (comparable)")

> print(results)

Variable Type Test p_value

1 cnsex Categorical Chi-square 0.6720438

2 hisei Continuous Wilcoxon 0.5827588

3 cnrandalt Continuous Wilcoxon 0.4456485

Interpretation

1 NOT significant (comparable)

2 NOT significant (comparable)

3 NOT significant (comparable)

**Project site, german proficiency (interpreter YES/NO), family structure (single parent), stress-HBS**

# Extract p-values

> p_cnsort <- chisq.test(table(dati_zeppelin$group, dati_zeppelin$cnsort))$p.value

> p_cnscre1 <- chisq.test(table(dati_zeppelin$group, dati_zeppelin$cnscre1))$p.value

> p_cnscre4b <- chisq.test(table(dati_zeppelin$group, dati_zeppelin$cnscre4b))$p.value

> # For continuous variable hbelges (using Wilcoxon as default)

> p_hbelges <- wilcox.test(hbelges ~ group, data = dati_zeppelin)$p.value

> # Create results table

> results <- data.frame(

+ Variable = c("cnsort", "cnscre1", "cnscre4b", "hbelges"),

+ Type = c("Categorical", "Dichotomous", "Dichotomous", "Continuous"),

+ Test = c("Chi-square", "Chi-square", "Chi-square", "Wilcoxon"),

+ p_value = c(p_cnsort, p_cnscre1, p_cnscre4b, p_hbelges)

+ )

> # Interpretation

> results$Interpretation <- ifelse(results$p_value < 0.05,

+ "SIGNIFICANT difference",

+ "NOT significant (comparable)")

> print(results)

Variable Type Test p_value

1 cnsort Categorical Chi-square 0.2145486

2 cnscre1 Dichotomous Chi-square 0.9124248

3 cnscre4b Dichotomous Chi-square 1.0000000

4 hbelges Continuous Wilcoxon 0.7368180

Interpretation

1 NOT significant (comparable)

2 NOT significant (comparable)

3 NOT significant (comparable)

4 NOT significant (comparable)

**cnrand (Types of randomization by stratification characteristics)**

> print(chisq_cnrand)

Pearson's Chi-squared test

data: tab_cnrand

X-squared = 1.4949, df = 6, p-value = 0.9598

> # Fisher's exact test if expected counts < 5

> fisher.test(tab_cnrand)

Fisher's Exact Test for Count Data

data: tab_cnrand

p-value = 0.9629

alternative hypothesis: two.sided

| > print(results)  Variable Type Test p_value  1 cnsort Categorical Chi-square 0.2145486  2 cnscre1 Dichotomous Chi-square 0.9124248  3 cnscre4b Dichotomous Chi-square 1.0000000  4 cnrand Categorical Chi-square 0.9598333  5 hbelges Continuous Wilcoxon 0.7368180  Interpretation  1 NOT significant (comparable)  2 NOT significant (comparable)  3 NOT significant (comparable)  4 NOT significant (comparable)  5 NOT significant (comparable) |
| --- |
|  |
| \|  \| \| --- \| |

1. **Handling of Missing Data**

**MICE imputation under MAR assumption**

| # ============================================================  > # MICE IMPUTATION  > # ============================================================  >  > # Step 1: Load libraries and raw data  > library(mice)  > library(dplyr)  >  > # Load raw data (with dummy codes -444, -555, etc.)  > raw_data <- read.csv("original dataset.csv")  >  > # ============================================================  > # Step 2: Convert dummy codes to NA  > # ============================================================  >  > dummy_codes <- c(-333, -444, -555, -777, -999)  >  > data_clean <- raw_data  >  > for(code in dummy_codes) {  + data_clean[data_clean == code] <- NA  + }  >  > # Check missing counts  > cat("Missing values after conversion:\n")  Missing values after conversion:  > missing_count <- colSums(is.na(data_clean))  > print(missing_count[missing_count > 0])  h3tage h3isei cngew e3part son5iq son5kon son5ver son5mot son5koo son5mon  3 16 1 22 20 20 20 20 20 20  >  > cat("\nTotal N:", nrow(data_clean), "\n")  Total N: 132  >  > # ============================================================  > # Step 2b: Calculate exact missing percentages  > # ============================================================  >  > missing_percent <- colSums(is.na(data_clean)) / nrow(data_clean) * 100  > missing_percent_sorted <- sort(missing_percent[missing_percent > 0], decreasing = TRUE)  >  > cat("=== MISSING PERCENTAGES ===\n")  === MISSING PERCENTAGES ===  > print(round(missing_percent_sorted, 1))  e3part son5iq son5kon son5ver son5mot son5koo son5mon h3isei h3tage cngew  16.7 15.2 15.2 15.2 15.2 15.2 15.2 12.1 2.3 0.8  >  > # Key variables  > key_vars <- c("e3part", "son5iq", "son5kon", "son5ver", "son5mot", "son5koo",  "son5mon", "h3isei")  >  > for(var in key_vars) {  + if(var %in% names(missing_percent_sorted)) {  + cat(var, ": ", round(missing_percent_sorted[var], 1), "%\n", sep = "")  + }  + }  e3part: 16.7%  son5iq: 15.2%  son5kon: 15.2%  son5ver: 15.2%  son5mot: 15.2%  son5koo: 15.2%  son5mon: 15.2%  h3isei: 12.1%  >  > # Overall missingness  > total_cells <- nrow(data_clean) * ncol(data_clean)  > missing_cells <- sum(is.na(data_clean))  > overall_missing_pct <- missing_cells / total_cells * 100  >  > cat("\n=== OVERALL MISSINGNESS ===\n")  === OVERALL MISSINGNESS ===  > cat("Total missing cells:", missing_cells, "out of", total_cells, "\n")  Total missing cells: 162 out of 2508  > cat("Overall missing percentage:", round(overall_missing_pct, 1), "%\n")  Overall missing percentage: 6.5 %  >  > # ============================================================  > # Step 3: Select variables for imputation  > # ============================================================  >  > data_impute <- data_clean %>%  + dplyr::select(cngrup, cnsex, h3tage, cnrandalt, h3isei, cngew,  + Europe_vs_Others, cnerr, logMeth, e3part,  + son5iq, son5kon, son5ver, son5mot, son5koo, son5mon)  >  > cat("\nVariables selected for imputation:\n")  Variables selected for imputation:  > print(names(data_impute))  [1] "cngrup" "cnsex" "h3tage" "cnrandalt"  "h3isei" "cngew"  [7] "Europe_vs_Others" "cnerr" "logMeth" "e3part"  "son5iq" "son5kon"  [13] "son5ver" "son5mot" "son5koo" "son5mon"  >  > # ============================================================  > # Step 4: Multiple Imputation with PMM (20 imputations)  > # ============================================================  >  > set.seed(2026)  >  > imp <- mice(data_impute,  + method = "pmm",  + m = 20,  + maxit = 20,  + printFlag = TRUE)  >  > # Save imputation object  > saveRDS(imp, file = "imputation_20_PMM_h3isei.rds")  >  > cat("\n=== IMPUTATION COMPLETE ===\n")  === IMPUTATION COMPLETE ===  > cat("Number of imputations:", imp$m, "\n")  Number of imputations: 20  > cat("Number of iterations:", imp$maxit, "\n")  Number of iterations:  >  > # ============================================================  > # Step 5: Create z-scores on imputed data  > # ============================================================  >  > imp_long <- complete(imp, action = "long", include = TRUE)  >  > add_z_scores <- function(data) {  +  + data$z_h3tage <- as.numeric(scale(data$h3tage))  + data$z_h3isei <- as.numeric(scale(data$h3isei)) # <--- CHANGED: was z_hisei  + data$z_logMeth <- as.numeric(scale(data$logMeth))  + data$z_e3part <- as.numeric(scale(data$e3part))  + data$z_son5iq <- as.numeric(scale(data$son5iq))  + data$z_son5kon <- as.numeric(scale(data$son5kon))  + data$z_son5ver <- as.numeric(scale(data$son5ver))  + data$z_son5mot <- as.numeric(scale(data$son5mot))  + data$z_son5koo <- as.numeric(scale(data$son5koo))  + data$z_son5mon <- as.numeric(scale(data$son5mon))  + data$z_cngew <- as.numeric(scale(data$cngew))  + data$z_cnrandalt <- as.numeric(scale(data$cnrandalt))  +  + return(data)  + }  >  > imp_transformed <- imp_long %>%  + group_by(.imp) %>%  + group_modify(~ as.data.frame(add_z_scores(.x))) %>%  + ungroup()  >  > imp_mids <- as.mids(imp_transformed)  >  > # Save transformed object  > saveRDS(imp_mids, file = "imputation_20_PMM_h3isei_transformed.rds")  >  > cat("\n=== TRANSFORMATION COMPLETE ===\n")  === TRANSFORMATION COMPLETE ===  > cat("Z-scores added for continuous/ordinal variables including z_h3isei\n")  Z-scores added for continuous/ordinal variables including z_h3isei  > cat("Categorical variables unchanged: cngrup, cnsex, Europe_vs_Others, cnerr\n")  Categorical variables unchanged: cngrup, cnsex, Europe_vs_Others, cnerr  >  >  **3. Regression Models (Models 1-4)**  >  > # ============================================================  > # MODELS 1-4: Linear Regressions  > # ============================================================  >  > library(mice)  > library(dplyr)  >  > imp <- readRDS("imputation_20_PMM_h3isei_transformed.rds")  >  > # ============================================================  > # MODEL 1: z_logMeth -> z_son5iq  > # Covariates: cnsex, Europe_vs_Others, z_h3isei, z_cngew  > # ============================================================  >  > model1 <- with(imp, lm(z_son5iq ~ z_logMeth + cnsex + Europe_vs_Others + z_h3isei +  z_cngew))  > pooled_model1 <- pool(model1)  > cat("\n========== MODEL 1 ==========\n")  ========== MODEL 1 ==========  > print(summary(pooled_model1, conf.int = FALSE))  term estimate std.error statistic df p.value  1 (Intercept) -0.06171207 0.19706178 -0.3131610 95.84985 0.75483874  2 z_logMeth -0.08373626 0.08600009 -0.9736764 111.57934 0.33232338  3 cnsex 0.34385157 0.17958014 1.9147527 96.17506 0.05849588  4 Europe_vs_Others -0.16396301 0.21338892 -0.7683764 83.56880 0.44442954  5 z_h3isei 0.23048415 0.09607416 2.3990233 81.64784 0.01871701  6 z_cngew 0.19165870 0.09099575 2.1062378 96.45630 0.03777987  >  > # ============================================================  > # MODEL 2: z_logMeth -> z_son5kon  > # Covariates: cnsex, Europe_vs_Others, z_h3isei, z_cngew, z_son5mon  > # ============================================================  >  > model2 <- with(imp, lm(z_son5kon ~ z_logMeth + cnsex + Europe_vs_Others + z_h3isei +  z_cngew + z_son5mon))  > pooled_model2 <- pool(model2)  > cat("\n========== MODEL 2 ==========\n")  ========== MODEL 2 ==========  > summary_df2 <- summary(pooled_model2, conf.int = FALSE)  > summary_df2$df <- round(summary_df2$df, 0)  > print(summary_df2)  term estimate std.error statistic df p.value  1 (Intercept) 0.202468530 0.19925532 1.01612607 104 0.311937278  2 z_logMeth 0.244052666 0.08564894 2.84945328 117 0.005174793  3 cnsex -0.438023739 0.18917770 -2.31540895 86 0.022967366  4 Europe_vs_Others 0.026495575 0.21268153 0.12457864 94 0.901124499  5 z_h3isei -0.108314688 0.09999338 -1.08321855 73 0.282263366  6 z_cngew 0.009856486 0.09216494 0.10694398 104 0.915039152  7 z_son5mon -0.005489188 0.09540601 -0.05753503 91 0.954244678  >  > # ============================================================  > # MODEL 3: z_logMeth -> z_son5ver, z_son5mot, z_son5koo  > # Covariates: cnsex, Europe_vs_Others, z_h3isei, z_cngew, z_son5mon  > # ============================================================  >  > # z_son5ver  > model3_ver <- with(imp, lm(z_son5ver ~ z_logMeth + cnsex + Europe_vs_Others + z_h3isei + z_cngew + z_son5mon))  > pooled_model3_ver <- pool(model3_ver)  > cat("\n========== MODEL 3: z_logMeth -> z_son5ver ==========\n")  ========== MODEL 3: z_logMeth -> z_son5ver ==========  > summary_df3_ver <- summary(pooled_model3_ver, conf.int = FALSE)  > summary_df3_ver$df <- round(summary_df3_ver$df, 0)  > print(summary_df3_ver)  term estimate std.error statistic df p.value  1 (Intercept) 0.09615772 0.21893301 0.4392107 81 0.66168391  2 z_logMeth 0.17078383 0.09040974 1.8889983 110 0.06151704  3 cnsex -0.09466906 0.19431349 -0.4871976 89 0.62731001  4 Europe_vs_Others -0.05671159 0.22223781 -0.2551843 90 0.79916210  5 z_h3isei -0.15166447 0.10210395 -1.4853929 78 0.14146661  6 z_cngew -0.08057250 0.10178347 -0.7916069 79 0.43095108  7 z_son5mon 0.06308821 0.09863203 0.6396321 92 0.52399984  >  > # z_son5mot  > model3_mot <- with(imp, lm(z_son5mot ~ z_logMeth + cnsex + Europe_vs_Others +  z_h3isei + z_cngew + z_son5mon))  > pooled_model3_mot <- pool(model3_mot)  > cat("\n========== MODEL 3: z_logMeth -> z_son5mot ==========\n")  ========== MODEL 3: z_logMeth -> z_son5mot ==========  > summary_df3_mot <- summary(pooled_model3_mot, conf.int = FALSE)  > summary_df3_mot$df <- round(summary_df3_mot$df, 0)  > print(summary_df3_mot)  term estimate std.error statistic df p.value  1 (Intercept) 0.23665569 0.20374755 1.1615143 104 0.2480912  2 z_logMeth 0.07288522 0.08858746 0.8227487 114 0.4123760  3 cnsex -0.10737169 0.19396117 -0.5535731 86 0.5813149  4 Europe_vs_Others -0.26327240 0.22531981 -1.1684388 81 0.2460776  5 z_h3isei -0.16434829 0.10425493 -1.5764079 68 0.1195973  6 z_cngew -0.03506680 0.09457116 -0.3707981 103 0.7115483  7 z_son5mon -0.02563186 0.10290267 -0.2490884 73 0.8039952  >  > # z_son5koo  > model3_koo <- with(imp, lm(z_son5koo ~ z_logMeth + cnsex + Europe_vs_Others +  z_h3isei + z_cngew + z_son5mon))  > pooled_model3_koo <- pool(model3_koo)  > cat("\n========== MODEL 3: z_logMeth -> z_son5koo ==========\n")  ========== MODEL 3: z_logMeth -> z_son5koo ==========  > summary_df3_koo <- summary(pooled_model3_koo, conf.int = FALSE)  > summary_df3_koo$df <- round(summary_df3_koo$df, 0)  > print(summary_df3_koo)  term estimate std.error statistic df p.value  1 (Intercept) 2.852497e-01 0.20273968 1.4069751705 108 0.16231392  2 z_logMeth 2.602287e-02 0.08863144 0.2936076404 115 0.76958751  3 cnsex -1.338104e-01 0.19217740 -0.6962859971 91 0.48802970  4 Europe_vs_Others -2.954740e-01 0.22722093 -1.3003817755 79 0.19724728  5 z_h3isei -1.678659e-01 0.09483928 -1.7700035649 104 0.07967101  6 z_cngew 5.582968e-05 0.09333581 0.0005981593 110 0.99952382  7 z_son5mon 6.394720e-02 0.10249788 0.6238880742 75 0.53458691  >  > # ============================================================  > # MODEL 4: cngrup -> z_logMeth  > # Covariates: cnsex, Europe_vs_Others, z_h3isei, z_cngew, z_h3tage, z_cnrandalt,  cnerr  > # ============================================================  >  > model4 <- with(imp, lm(z_logMeth ~ cngrup + cnsex + Europe_vs_Others + z_h3isei +  z_cngew +  + z_h3tage + z_cnrandalt + cnerr))  > pooled_model4 <- pool(model4)  > cat("\n========== MODEL 4 ==========\n")  ========== MODEL 4 ==========  > summary_df4 <- summary(pooled_model4, conf.int = FALSE)  > summary_df4$df <- round(summary_df4$df, 0)  > print(summary_df4)  term estimate std.error statistic df p.value  1 (Intercept) -0.34762859 0.30865789 -1.1262586 121 0.262287301  2 cngrup -0.37473970 0.18118304 -2.0682935 121 0.040743831  3 cnsex 0.15524601 0.17318268 0.8964292 121 0.371803560  4 Europe_vs_Others 0.07975133 0.20309818 0.3926738 121 0.695252850  5 z_h3isei -0.05535785 0.09315197 -0.5942747 116 0.553486272  6 z_cngew -0.04508678 0.08871194 -0.5082380 121 0.612213925  7 z_h3tage -0.13816032 0.08983447 -1.5379433 121 0.126677594  8 z_cnrandalt 0.23679854 0.08968717 2.6402723 121 0.009376625  9 cnerr 0.46509145 0.26989974 1.7232008 121 0.087406706  >  **4. Mediation Models (Models 5-7)**  > # ============================================================  > # MODELS 5-7: Simple Mediations  > # ============================================================  >  > library(mice)  > library(mediation)  >  > # Load transformed dataset  > imp <- readRDS("imputation_20_PMM_h3isei_transformed.rds")  >  > # ============================================================  > # MODEL 5: cngrup -> z_logMeth -> z_son5kon  > # ============================================================  >  > run_mediation5 <- function(data) {  + med_model <- lm(z_logMeth ~ cngrup + cnerr + z_cnrandalt + z_h3tage +  + cnsex + Europe_vs_Others + z_cngew + z_h3isei,  + data = data)  + out_model <- lm(z_son5kon ~ cngrup + z_logMeth + cnerr + z_cnrandalt +  + cnsex + z_son5mon + Europe_vs_Others + z_h3isei + z_cngew,  + data = data)  + result <- mediate(med_model, out_model, treat = "cngrup", mediator = "z_logMeth",  + sims = 500, boot = TRUE)  + data.frame(acme = result$d0, ade = result$z0, total = result$tau.coef,  + prop = result$n0, p_acme = result$d0.p, p_ade = result$z0.p)  + }  >  > set.seed(2026)  > results_list5 <- list()  > for(i in 1:imp$m) {  + cat("Processing imputation", i, "of", imp$m, " (Model 5)\n")  + data_i <- complete(imp, action = i)  + results_list5[[i]] <- run_mediation5(data_i)  + }  Processing imputation 1 of 20 (Model 5)  Running nonparametric bootstrapProcessing imputation 2 of 20 (Model 5)  Running nonparametric bootstrapProcessing imputation 3 of 20 (Model 5)  Running nonparametric bootstrapProcessing imputation 4 of 20 (Model 5)  Running nonparametric bootstrapProcessing imputation 5 of 20 (Model 5)  Running nonparametric bootstrapProcessing imputation 6 of 20 (Model 5)  Running nonparametric bootstrapProcessing imputation 7 of 20 (Model 5)  Running nonparametric bootstrapProcessing imputation 8 of 20 (Model 5)  Running nonparametric bootstrapProcessing imputation 9 of 20 (Model 5)  Running nonparametric bootstrapProcessing imputation 10 of 20 (Model 5)  Running nonparametric bootstrapProcessing imputation 11 of 20 (Model 5)  Running nonparametric bootstrapProcessing imputation 12 of 20 (Model 5)  Running nonparametric bootstrapProcessing imputation 13 of 20 (Model 5)  Running nonparametric bootstrapProcessing imputation 14 of 20 (Model 5)  Running nonparametric bootstrapProcessing imputation 15 of 20 (Model 5)  Running nonparametric bootstrapProcessing imputation 16 of 20 (Model 5)  Running nonparametric bootstrapProcessing imputation 17 of 20 (Model 5)  Running nonparametric bootstrapProcessing imputation 18 of 20 (Model 5)  Running nonparametric bootstrapProcessing imputation 19 of 20 (Model 5)  Running nonparametric bootstrapProcessing imputation 20 of 20 (Model 5)  Running nonparametric bootstrap> all_results5 <- do.call(rbind, results_list5)  >  > cat("\n========== MODEL 5: cngrup -> z_logMeth -> z_son5kon ==========\n")  ========== MODEL 5: cngrup -> z_logMeth -> z_son5kon ==========  > cat("Indirect Effect (ACME):", round(mean(all_results5$acme), 3),  + "p =", round(mean(all_results5$p_acme), 4), "\n")  Indirect Effect (ACME): -0.085 p = 0.0866  > cat("Direct Effect (ADE):", round(mean(all_results5$ade), 3),  + "p =", round(mean(all_results5$p_ade), 4), "\n")  Direct Effect (ADE): -0.025 p = 0.7582  > cat("Total Effect:", round(mean(all_results5$total), 3), "\n")  Total Effect: -0.11  > cat("Proportion Mediated:", round(mean(all_results5$prop), 3), "\n")  Proportion Mediated: 1.47  >  > # ============================================================  > # MODEL 6: z_logMeth -> z_son5kon -> z_son5iq  > # ============================================================  >  > run_mediation6 <- function(data) {  + med_model <- lm(z_son5kon ~ z_logMeth + z_h3tage + cnsex +  + Europe_vs_Others + z_cngew + z_h3isei + z_son5mon,  + data = data)  + out_model <- lm(z_son5iq ~ z_logMeth + z_son5kon + cnsex +  + Europe_vs_Others + z_h3isei + z_cngew,  + data = data)  + result <- mediate(med_model, out_model, treat = "z_logMeth", mediator = "z_son5kon",  + sims = 500, boot = TRUE)  + data.frame(acme = result$d0, ade = result$z0, total = result$tau.coef,  + prop = result$n0, p_acme = result$d0.p, p_ade = result$z0.p)  + }  >  > set.seed(2026)  > results_list6 <- list()  > for(i in 1:imp$m) {  + cat("Processing imputation", i, "of", imp$m, " (Model 6)\n")  + data_i <- complete(imp, action = i)  + results_list6[[i]] <- run_mediation6(data_i)  + }  Processing imputation 1 of 20 (Model 6)  Running nonparametric bootstrapProcessing imputation 2 of 20 (Model 6)  Running nonparametric bootstrapProcessing imputation 3 of 20 (Model 6)  Running nonparametric bootstrapProcessing imputation 4 of 20 (Model 6)  Running nonparametric bootstrapProcessing imputation 5 of 20 (Model 6)  Running nonparametric bootstrapProcessing imputation 6 of 20 (Model 6)  Running nonparametric bootstrapProcessing imputation 7 of 20 (Model 6)  Running nonparametric bootstrapProcessing imputation 8 of 20 (Model 6)  Running nonparametric bootstrapProcessing imputation 9 of 20 (Model 6)  Running nonparametric bootstrapProcessing imputation 10 of 20 (Model 6)  Running nonparametric bootstrapProcessing imputation 11 of 20 (Model 6)  Running nonparametric bootstrapProcessing imputation 12 of 20 (Model 6)  Running nonparametric bootstrapProcessing imputation 13 of 20 (Model 6)  Running nonparametric bootstrapProcessing imputation 14 of 20 (Model 6)  Running nonparametric bootstrapProcessing imputation 15 of 20 (Model 6)  Running nonparametric bootstrapProcessing imputation 16 of 20 (Model 6)  Running nonparametric bootstrapProcessing imputation 17 of 20 (Model 6)  Running nonparametric bootstrapProcessing imputation 18 of 20 (Model 6)  Running nonparametric bootstrapProcessing imputation 19 of 20 (Model 6)  Running nonparametric bootstrapProcessing imputation 20 of 20 (Model 6)  Running nonparametric bootstrap> all_results6 <- do.call(rbind, results_list6)  >  > cat("\n========== MODEL 6: z_logMeth -> z_son5kon -> z_son5iq ==========\n")  ========== MODEL 6: z_logMeth -> z_son5kon -> z_son5iq ==========  > cat("Indirect Effect (ACME):", round(mean(all_results6$acme), 3),  + "p =", round(mean(all_results6$p_acme), 4), "\n")  Indirect Effect (ACME): -0.083 p = 0.0124  > cat("Direct Effect (ADE):", round(mean(all_results6$ade), 3),  + "p =", round(mean(all_results6$p_ade), 4), "\n")  Direct Effect (ADE): 0.004 p = 0.8024  > cat("Total Effect:", round(mean(all_results6$total), 3), "\n")  Total Effect: -0.079  > cat("Proportion Mediated:", round(mean(all_results6$prop), 3), "\n")  Proportion Mediated: 1.151  >  > # ============================================================  > # MODEL 7: z_e3part_rev -> z_logMeth -> z_son5kon  > # (Create z_e3part_rev directly from transformed data)  > # ============================================================  >  > library(mice)  > library(mediation)  >  > # Load transformed dataset (with z-scores)  > imp <- readRDS("imputation_20_PMM_h3isei_transformed.rds")  >  > # ============================================================  > # Create z_e3part_rev on the fly  > # ============================================================  >  > # Extract long format and create reversed variable  > imp_long <- complete(imp, action = "long", include = TRUE)  >  > # Reverse e3part: higher values = more disagreement  > imp_long$z_e3part_rev <- -imp_long$z_e3part  >  > # Convert back to mids object  > imp_rev <- as.mids(imp_long)  >  > # Optional: save it for later use  > saveRDS(imp_rev, file = "imputation_20_PMM_h3isei_reversed.rds")  >  > # ============================================================  > # Run Mediation Model 7  > # ============================================================  >  > run_mediation7 <- function(data) {  +  + med_model <- lm(z_logMeth ~ z_e3part_rev + z_h3tage + cnsex +  + Europe_vs_Others + z_cngew + z_h3isei,  + data = data)  +  + out_model <- lm(z_son5kon ~ z_e3part_rev + z_logMeth + cnsex +  + z_son5mon + Europe_vs_Others + z_h3isei + z_cngew,  + data = data)  +  + result <- mediate(med_model, out_model,  + treat = "z_e3part_rev",  + mediator = "z_logMeth",  + sims = 500,  + boot = TRUE)  +  + data.frame(  + acme = result$d0,  + ade = result$z0,  + total = result$tau.coef,  + prop = result$n0,  + p_acme = result$d0.p,  + p_ade = result$z0.p  + )  + }  >  > # Run on all imputations  > set.seed(2026)  > results_list7 <- list()  >  > for(i in 1:imp_rev$m) {  + cat("Processing imputation", i, "of", imp_rev$m, " (Model 7)\n")  + data_i <- complete(imp_rev, action = i)  + results_list7[[i]] <- run_mediation7(data_i)  + }  Processing imputation 1 of 20 (Model 7)  Running nonparametric bootstrapProcessing imputation 2 of 20 (Model 7)  Running nonparametric bootstrapProcessing imputation 3 of 20 (Model 7)  Running nonparametric bootstrapProcessing imputation 4 of 20 (Model 7)  Running nonparametric bootstrapProcessing imputation 5 of 20 (Model 7)  Running nonparametric bootstrapProcessing imputation 6 of 20 (Model 7)  Running nonparametric bootstrapProcessing imputation 7 of 20 (Model 7)  Running nonparametric bootstrapProcessing imputation 8 of 20 (Model 7)  Running nonparametric bootstrapProcessing imputation 9 of 20 (Model 7)  Running nonparametric bootstrapProcessing imputation 10 of 20 (Model 7)  Running nonparametric bootstrapProcessing imputation 11 of 20 (Model 7)  Running nonparametric bootstrapProcessing imputation 12 of 20 (Model 7)  Running nonparametric bootstrapProcessing imputation 13 of 20 (Model 7)  Running nonparametric bootstrapProcessing imputation 14 of 20 (Model 7)  Running nonparametric bootstrapProcessing imputation 15 of 20 (Model 7)  Running nonparametric bootstrapProcessing imputation 16 of 20 (Model 7)  Running nonparametric bootstrapProcessing imputation 17 of 20 (Model 7)  Running nonparametric bootstrapProcessing imputation 18 of 20 (Model 7)  Running nonparametric bootstrapProcessing imputation 19 of 20 (Model 7)  Running nonparametric bootstrapProcessing imputation 20 of 20 (Model 7)  Running nonparametric bootstrap>  > # Combine and pool results  > all_results7 <- do.call(rbind, results_list7)  >  > cat("\n========== MODEL 7: z_e3part_rev -> z_logMeth -> z_son5kon ==========\n")  ========== MODEL 7: z_e3part_rev -> z_logMeth -> z_son5kon ==========  > cat("Indirect Effect (ACME):", round(mean(all_results7$acme), 3),  + " (SD =", round(sd(all_results7$acme), 3), ")\n")  Indirect Effect (ACME): 0.064 (SD = 0.007 )  > cat("p-value:", round(mean(all_results7$p_acme), 4), "\n\n")  p-value: 0.023  > cat("Direct Effect (ADE):", round(mean(all_results7$ade), 3),  + " (SD =", round(sd(all_results7$ade), 3), ")\n")  Direct Effect (ADE): 0.051 (SD = 0.05 )  > cat("p-value:", round(mean(all_results7$p_ade), 4), "\n\n")  p-value: 0.6024  > cat("Total Effect:", round(mean(all_results7$total), 3), "\n")  Total Effect: 0.115  > cat("Proportion Mediated:", round(mean(all_results7$prop), 3), "\n")  Proportion Mediated: 0.677  **5. Moderated and Serial Mediation Models (Models 8-10)**  > # ============================================================  > # MODEL 8: Moderated Mediation (IG vs CG)  > # z_e3part_rev -> z_logMeth -> z_son5kon  > # ============================================================  >  > library(mice)  > library(mediation)  >  > # Load transformed dataset and create imp_rev (if not already in memory)  > imp <- readRDS("imputation_20_PMM_h3isei_transformed.rds")  >  > imp_long <- complete(imp, action = "long", include = TRUE)  > imp_long$z_e3part_rev <- -imp_long$z_e3part  > imp_rev <- as.mids(imp_long)  >  > # ============================================================  > # Function for one group  > # ============================================================  >  > run_mediation_group <- function(data, group_value) {  +  + data_group <- data[data$cngrup == group_value, ]  +  + if(nrow(data_group) < 20) {  + return(data.frame(acme = NA, p_acme = NA, n = nrow(data_group)))  + }  +  + med_model <- lm(z_logMeth ~ z_e3part_rev + z_h3tage + cnsex +  + Europe_vs_Others + z_cngew + z_h3isei + cnerr + z_cnrandalt,  + data = data_group)  +  + out_model <- lm(z_son5kon ~ z_e3part_rev + z_logMeth + cnsex +  + z_son5mon + Europe_vs_Others + z_h3isei + z_cngew,  + data = data_group)  +  + result <- mediate(med_model, out_model,  + treat = "z_e3part_rev",  + mediator = "z_logMeth",  + sims = 200,  + boot = TRUE)  +  + return(data.frame(acme = result$d0, p_acme = result$d0.p, n = nrow(data_group)))  + }  >  > # ============================================================  > # Run on all imputations  > # ============================================================  >  > set.seed(2026)  >  > results_cg <- list()  > results_ig <- list()  >  > for(i in 1:imp_rev$m) {  + cat("Imputation", i, "of", imp_rev$m, "\n")  + data_i <- complete(imp_rev, action = i)  +  + results_cg[[i]] <- run_mediation_group(data_i, group_value = 0)  + results_ig[[i]] <- run_mediation_group(data_i, group_value = 1)  + }  Imputation 1 of 20  Running nonparametric bootstrap  Running nonparametric bootstrapImputation 2 of 20  Running nonparametric bootstrap  Running nonparametric bootstrapImputation 3 of 20  Running nonparametric bootstrap  Running nonparametric bootstrapImputation 4 of 20  Running nonparametric bootstrap  Running nonparametric bootstrapImputation 5 of 20  Running nonparametric bootstrap  Running nonparametric bootstrapImputation 6 of 20  Running nonparametric bootstrap  Running nonparametric bootstrapImputation 7 of 20  Running nonparametric bootstrap  Running nonparametric bootstrapImputation 8 of 20  Running nonparametric bootstrap  Running nonparametric bootstrapImputation 9 of 20  Running nonparametric bootstrap  Running nonparametric bootstrapImputation 10 of 20  Running nonparametric bootstrap  Running nonparametric bootstrapImputation 11 of 20  Running nonparametric bootstrap  Running nonparametric bootstrapImputation 12 of 20  Running nonparametric bootstrap  Running nonparametric bootstrapImputation 13 of 20  Running nonparametric bootstrap  Running nonparametric bootstrapImputation 14 of 20  Running nonparametric bootstrap  Running nonparametric bootstrapImputation 15 of 20  Running nonparametric bootstrap  Running nonparametric bootstrapImputation 16 of 20  Running nonparametric bootstrap  Running nonparametric bootstrapImputation 17 of 20  Running nonparametric bootstrap  Running nonparametric bootstrapImputation 18 of 20  Running nonparametric bootstrap  Running nonparametric bootstrapImputation 19 of 20  Running nonparametric bootstrap  Running nonparametric bootstrapImputation 20 of 20  Running nonparametric bootstrap  Running nonparametric bootstrap  There were 50 or more warnings (use warnings() to see the first 50)>  > # ============================================================  > # Combine and pool results  > # ============================================================  >  > cg_all <- do.call(rbind, results_cg)  > ig_all <- do.call(rbind, results_ig)  >  > # Remove NA  > cg_clean <- cg_all[!is.na(cg_all$acme), ]  > ig_clean <- ig_all[!is.na(ig_all$acme), ]  >  > # Control Group  > pooled_acme_cg <- mean(cg_clean$acme)  > pooled_p_cg <- mean(cg_clean$p_acme)  > sd_acme_cg <- sd(cg_clean$acme)  > n_cg <- round(mean(cg_clean$n))  >  > # Intervention Group  > pooled_acme_ig <- mean(ig_clean$acme)  > pooled_p_ig <- mean(ig_clean$p_acme)  > sd_acme_ig <- sd(ig_clean$acme)  > n_ig <- round(mean(ig_clean$n))  >  > # Difference  > diff_acme <- pooled_acme_ig - pooled_acme_cg  > se_diff <- sqrt(sd_acme_cg^2 + sd_acme_ig^2)  > z_diff <- diff_acme / se_diff  > p_diff <- 2 * (1 - pnorm(abs(z_diff)))  >  > # ============================================================  > # Print results  > # ============================================================  >  > cat("\n============================================================\n")  ============================================================  > cat("MODEL 8: Moderated Mediation\n")  MODEL 8: Moderated Mediation  > cat("z_e3part_rev -> z_logMeth -> z_son5kon\n")  z_e3part_rev -> z_logMeth -> z_son5kon  > cat("============================================================\n\n")  ============================================================  >  > cat("Control Group (CG):\n")  Control Group (CG):  > cat(" N:", n_cg, "\n")  N: 60  > cat(" Indirect effect:", round(pooled_acme_cg, 4), "\n")  Indirect effect: 0.0803  > cat(" SD:", round(sd_acme_cg, 4), "\n")  SD: 0.0252  > cat(" p-value:", round(pooled_p_cg, 4), "\n\n")  p-value: 0.265  >  > cat("Intervention Group (IG):\n")  Intervention Group (IG):  > cat(" N:", n_ig, "\n")  N: 72  > cat(" Indirect effect:", round(pooled_acme_ig, 4), "\n")  Indirect effect: 0.0639  > cat(" SD:", round(sd_acme_ig, 4), "\n")  SD: 0.0068  > cat(" p-value:", round(pooled_p_ig, 4), "\n\n")  p-value: 0.0875  >  > cat("Difference (IG - CG):", round(diff_acme, 4), "\n")  Difference (IG - CG): -0.0164  > cat(" SE:", round(se_diff, 4), "\n")  SE: 0.0261  > cat(" z-value:", round(z_diff, 2), "\n")  z-value: -0.63  > cat(" p-value:", round(p_diff, 4), "\n\n")  p-value: 0.531  >  > if(p_diff < 0.05) {  + cat("✓ Significant moderation\n")  + } else {  + cat("✗ No significant moderation\n")  + }  ✗ No significant moderation  >  > # ============================================================  > # Save results  > # ============================================================  >  > group_results <- data.frame(  + Group = c("Control (CG)", "Intervention (IG)"),  + N = c(n_cg, n_ig),  + Indirect_Effect = c(pooled_acme_cg, pooled_acme_ig),  + SD = c(sd_acme_cg, sd_acme_ig),  + p_value = c(pooled_p_cg, pooled_p_ig)  + )  >  > write.csv(group_results, "model8_moderated_mediation_h3isei.csv", row.names = FALSE)  >  > cat("\nResults saved to model8_moderated_mediation_h3isei.csv\n")  Results saved to model8_moderated_mediation_h3isei.csv  >  >  > # ============================================================  > # MODEL 9: Serial Mediation  > # z_e3part_rev -> z_logMeth -> z_son5kon -> z_son5iq  > # ============================================================  >  > library(mice)  > library(boot)  >  > # Load transformed dataset and create imp_rev (if not already in memory)  > imp <- readRDS("imputation_20_PMM_h3isei_transformed.rds")  >  > imp_long <- complete(imp, action = "long", include = TRUE)  > imp_long$z_e3part_rev <- -imp_long$z_e3part  > imp_rev <- as.mids(imp_long)  >  > # ============================================================  > # Function to run serial mediation on one dataset  > # ============================================================  >  > run_serial_mediation <- function(data) {  +  + # Path a: z_e3part_rev -> z_logMeth  + model_a <- lm(z_logMeth ~ z_e3part_rev + cnsex + z_h3tage +  + Europe_vs_Others + z_cngew + z_h3isei,  + data = data)  + a_coef <- coef(model_a)["z_e3part_rev"]  +  + # Path b1: z_logMeth -> z_son5kon  + model_b1 <- lm(z_son5kon ~ z_logMeth + cnsex + z_son5mon +  + Europe_vs_Others + z_cngew + z_h3isei,  + data = data)  + b1_coef <- coef(model_b1)["z_logMeth"]  +  + # Path b2: z_son5kon -> z_son5iq  + model_b2 <- lm(z_son5iq ~ z_son5kon + cnsex + Europe_vs_Others +  + z_cngew + z_h3isei,  + data = data)  + b2_coef <- coef(model_b2)["z_son5kon"]  +  + # Serial indirect effect  + ab_serial <- a_coef * b1_coef * b2_coef  +  + # Bootstrap for standard error  + boot_serial <- function(d, indices) {  + d_sub <- d[indices, ]  + a_boot <- coef(lm(z_logMeth ~ z_e3part_rev + cnsex + z_h3tage +  + Europe_vs_Others + z_cngew + z_h3isei, data = d_sub))  ["z_e3part_rev"]  + b1_boot <- coef(lm(z_son5kon ~ z_logMeth + cnsex + z_son5mon +  + Europe_vs_Others + z_cngew + z_h3isei, data = d_sub))  ["z_logMeth"]  + b2_boot <- coef(lm(z_son5iq ~ z_son5kon + cnsex + Europe_vs_Others +  + z_cngew + z_h3isei, data = d_sub))["z_son5kon"]  + return(a_boot * b1_boot * b2_boot)  + }  +  + boot_result <- boot::boot(data, boot_serial, R = 200)  + boot_se <- sd(boot_result$t, na.rm = TRUE)  + p_serial <- 2 * (1 - pnorm(abs(ab_serial / boot_se)))  +  + return(data.frame(  + a = a_coef,  + b1 = b1_coef,  + b2 = b2_coef,  + ab_serial = ab_serial,  + boot_se = boot_se,  + p = p_serial  + ))  + }  >  > # ============================================================  > # Run on all imputations  > # ============================================================  >  > set.seed(2026)  > serial_results <- list()  >  > for(i in 1:imp_rev$m) {  + cat("Processing imputation", i, "of", imp_rev$m, " (Model 9)\n")  + data_i <- complete(imp_rev, action = i)  + serial_results[[i]] <- run_serial_mediation(data_i)  + }  Processing imputation 1 of 20 (Model 9)  Processing imputation 2 of 20 (Model 9)  Processing imputation 3 of 20 (Model 9)  Processing imputation 4 of 20 (Model 9)  Processing imputation 5 of 20 (Model 9)  Processing imputation 6 of 20 (Model 9)  Processing imputation 7 of 20 (Model 9)  Processing imputation 8 of 20 (Model 9)  Processing imputation 9 of 20 (Model 9)  Processing imputation 10 of 20 (Model 9)  Processing imputation 11 of 20 (Model 9)  Processing imputation 12 of 20 (Model 9)  Processing imputation 13 of 20 (Model 9)  Processing imputation 14 of 20 (Model 9)  Processing imputation 15 of 20 (Model 9)  Processing imputation 16 of 20 (Model 9)  Processing imputation 17 of 20 (Model 9)  Processing imputation 18 of 20 (Model 9)  Processing imputation 19 of 20 (Model 9)  Processing imputation 20 of 20 (Model 9)  >  > # Combine results  > all_serial <- do.call(rbind, serial_results)  >  > # ============================================================  > # Print results  > # ============================================================  >  > cat("\n============================================================\n")  ============================================================  > cat("MODEL 9: Serial Mediation\n")  MODEL 9: Serial Mediation  > cat("z_e3part_rev -> z_logMeth -> z_son5kon -> z_son5iq\n")  z_e3part_rev -> z_logMeth -> z_son5kon -> z_son5iq  > cat("============================================================\n\n")  ============================================================  >  > cat("Path a (z_e3part_rev -> z_logMeth):", round(mean(all_serial$a), 4), "\n")  Path a (z_e3part_rev -> z_logMeth): 0.276  > cat("Path b1 (z_logMeth -> z_son5kon):", round(mean(all_serial$b1), 4), "\n")  Path b1 (z_logMeth -> z_son5kon): 0.2441  > cat("Path b2 (z_son5kon -> z_son5iq):", round(mean(all_serial$b2), 4), "\n\n")  Path b2 (z_son5kon -> z_son5iq): -0.3583  >  > cat("Serial Indirect Effect (a * b1 * b2):\n")  Serial Indirect Effect (a * b1 * b2):  > cat(" Estimate:", round(mean(all_serial$ab_serial), 4), "\n")  Estimate: -0.0241  > cat(" Boot SE:", round(mean(all_serial$boot_se), 4), "\n")  Boot SE: 0.0152  > cat(" p-value:", round(mean(all_serial$p), 4), "\n\n")  p-value: 0.119  >  > if(mean(all_serial$p) < 0.05) {  + cat("✓ The serial mediation effect is significant.\n")  + } else {  + cat("✗ The serial mediation effect is not significant.\n")  + }  ✗ The serial mediation effect is not significant.  >  > # ============================================================  > # Save results  > # ============================================================  >  > write.csv(all_serial, "model9_serial_mediation_h3isei.csv", row.names = FALSE)  >  > cat("\nResults saved to model9_serial_mediation_h3isei.csv\n")  Results saved to model9_serial_mediation_h3isei.csv  >  >  > # ============================================================  > # MODEL 10: Moderated Serial Mediation (IG vs CG)  > # z_e3part_rev -> z_logMeth -> z_son5kon -> z_son5iq  > # ============================================================  >  > library(mice)  > library(boot)  >  > # Load transformed dataset and create imp_rev (if not already in memory)  > imp <- readRDS("imputation_20_PMM_h3isei_transformed.rds")  >  > imp_long <- complete(imp, action = "long", include = TRUE)  > imp_long$z_e3part_rev <- -imp_long$z_e3part  > imp_rev <- as.mids(imp_long)  >  > # ============================================================  > # Function for one group  > # ============================================================  >  > run_serial_group <- function(data, group_value, group_name) {  +  + data_group <- data[data$cngrup == group_value, ]  +  + cat(" ", group_name, "N =", nrow(data_group), "\n")  +  + if(nrow(data_group) < 20) {  + return(data.frame(ab_serial = NA, p = NA, n = nrow(data_group)))  + }  +  + # Path a  + model_a <- lm(z_logMeth ~ z_e3part_rev + cnsex + z_h3tage +  + Europe_vs_Others + z_cngew + z_h3isei,  + data = data_group)  + a_coef <- coef(model_a)["z_e3part_rev"]  +  + # Path b1  + model_b1 <- lm(z_son5kon ~ z_logMeth + cnsex + z_son5mon +  + Europe_vs_Others + z_cngew + z_h3isei,  + data = data_group)  + b1_coef <- coef(model_b1)["z_logMeth"]  +  + # Path b2  + model_b2 <- lm(z_son5iq ~ z_son5kon + cnsex + Europe_vs_Others +  + z_cngew + z_h3isei,  + data = data_group)  + b2_coef <- coef(model_b2)["z_son5kon"]  +  + # Serial indirect  + ab_serial <- a_coef * b1_coef * b2_coef  +  + # Bootstrap  + boot_serial <- function(d, indices) {  + d_sub <- d[indices, ]  + a_boot <- coef(lm(z_logMeth ~ z_e3part_rev + cnsex + z_h3tage +  + Europe_vs_Others + z_cngew + z_h3isei, data = d_sub))  ["z_e3part_rev"]  + b1_boot <- coef(lm(z_son5kon ~ z_logMeth + cnsex + z_son5mon +  + Europe_vs_Others + z_cngew + z_h3isei, data = d_sub))  ["z_logMeth"]  + b2_boot <- coef(lm(z_son5iq ~ z_son5kon + cnsex + Europe_vs_Others +  + z_cngew + z_h3isei, data = d_sub))["z_son5kon"]  + return(a_boot * b1_boot * b2_boot)  + }  +  + boot_result <- tryCatch({  + boot::boot(data_group, boot_serial, R = 200)  + }, error = function(e) return(NULL))  +  + if(!is.null(boot_result)) {  + boot_se <- sd(boot_result$t, na.rm = TRUE)  + p_serial <- 2 * (1 - pnorm(abs(ab_serial / boot_se)))  + } else {  + p_serial <- NA  + }  +  + return(data.frame(  + group = group_name,  + n = nrow(data_group),  + ab_serial = ab_serial,  + p = p_serial  + ))  + }  >  > # ============================================================  > # Run on all imputations  > # ============================================================  >  > set.seed(2026)  >  > results_cg <- list()  > results_ig <- list()  >  > for(i in 1:imp_rev$m) {  + cat("\nImputation", i, "of", imp_rev$m, "\n")  + data_i <- complete(imp_rev, action = i)  +  + results_cg[[i]] <- run_serial_group(data_i, group_value = 0, group_name = "CG")  + results_ig[[i]] <- run_serial_group(data_i, group_value = 1, group_name = "IG")  + }  Imputation 1 of 20  CG N = 60  IG N = 72  Imputation 2 of 20  CG N = 60  IG N = 72  Imputation 3 of 20  CG N = 60  IG N = 72  Imputation 4 of 20  CG N = 60  IG N = 72  Imputation 5 of 20  CG N = 60  IG N = 72  Imputation 6 of 20  CG N = 60  IG N = 72  Imputation 7 of 20  CG N = 60  IG N = 72  Imputation 8 of 20  CG N = 60  IG N = 72  Imputation 9 of 20  CG N = 60  IG N = 72  Imputation 10 of 20  CG N = 60  IG N = 72  Imputation 11 of 20  CG N = 60  IG N = 72  Imputation 12 of 20  CG N = 60  IG N = 72  Imputation 13 of 20  CG N = 60  IG N = 72  Imputation 14 of 20  CG N = 60  IG N = 72  Imputation 15 of 20  CG N = 60  IG N = 72  Imputation 16 of 20  CG N = 60  IG N = 72  Imputation 17 of 20  CG N = 60  IG N = 72  Imputation 18 of 20  CG N = 60  IG N = 72  Imputation 19 of 20  CG N = 60  IG N = 72  Imputation 20 of 20  CG N = 60  IG N = 72  >  > # ============================================================  > # Combine results  > # ============================================================  >  > cg_all <- do.call(rbind, results_cg)  > ig_all <- do.call(rbind, results_ig)  >  > # Remove NA  > cg_clean <- cg_all[!is.na(cg_all$ab_serial), ]  > ig_clean <- ig_all[!is.na(ig_all$ab_serial), ]  >  > # Pool within groups  > ab_cg <- mean(cg_clean$ab_serial)  > p_cg <- mean(cg_clean$p)  > n_cg <- round(mean(cg_clean$n))  > sd_cg <- sd(cg_clean$ab_serial)  >  > ab_ig <- mean(ig_clean$ab_serial)  > p_ig <- mean(ig_clean$p)  > n_ig <- round(mean(ig_clean$n))  > sd_ig <- sd(ig_clean$ab_serial)  >  > # Difference between groups  > diff_serial <- ab_ig - ab_cg  > se_diff <- sqrt(sd_cg^2 + sd_ig^2)  > z_diff <- diff_serial / se_diff  > p_diff <- 2 * (1 - pnorm(abs(z_diff)))  >  > # ============================================================  > # Print results  > # ============================================================  >  > cat("\n============================================================\n")  ============================================================  > cat("MODEL 10: Moderated Serial Mediation\n")  MODEL 10: Moderated Serial Mediation  > cat("z_e3part_rev -> z_logMeth -> z_son5kon -> z_son5iq\n")  z_e3part_rev -> z_logMeth -> z_son5kon -> z_son5iq  > cat("============================================================\n\n")  ============================================================  >  > cat("Control Group (CG):\n")  Control Group (CG):  > cat(" N:", n_cg, "\n")  N: 60  > cat(" Serial indirect effect:", round(ab_cg, 4), "\n")  Serial indirect effect: -0.0298  > cat(" SD:", round(sd_cg, 4), "\n")  SD: 0.0056  > cat(" p-value:", round(p_cg, 4), "\n\n")  p-value: 0.347  >  > cat("Intervention Group (IG):\n")  Intervention Group (IG):  > cat(" N:", n_ig, "\n")  N: 72  > cat(" Serial indirect effect:", round(ab_ig, 4), "\n")  Serial indirect effect: -0.019  > cat(" SD:", round(sd_ig, 4), "\n")  SD: 0.0032  > cat(" p-value:", round(p_ig, 4), "\n\n")  p-value: 0.2145  >  > cat("Difference (IG - CG):", round(diff_serial, 4), "\n")  Difference (IG - CG): 0.0108  > cat(" SE:", round(se_diff, 4), "\n")  SE: 0.0064  > cat(" z-value:", round(z_diff, 2), "\n")  z-value: 1.67  > cat(" p-value:", round(p_diff, 4), "\n\n")  p-value: 0.0953  >  > if(p_diff < 0.05) {  + cat("✓ Significant moderation: The serial mediation effect differs between groups  .\n")  + } else {  + cat("✗ No significant moderation: The serial mediation effect does not differ  between groups.\n")  + }  ✗ No significant moderation: The serial mediation effect does not differ between  groups. |
| --- |
|  |

**6. Sensitivity Analyses (Complete Cases & NMAR)**

**Complete cases analyses**

# ============================================================

> # COMPLETE CASES ANALYSIS (NO IMPUTATION)

> # ============================================================

>

> library(dplyr)

> library(mediation)

> library(boot)

>

> # Load raw data

> raw_data <- read.csv("original dataset.csv")

>

> # ============================================================

> # Step 1: Convert dummy codes to NA

> # ============================================================

>

> dummy_codes <- c(-333, -444, -555, -777, -999)

>

> data_clean <- raw_data

>

> for(code in dummy_codes) {

+ data_clean[data_clean == code] <- NA

+ }

>

> # ============================================================

> # Step 2: Select variables and create complete cases dataset

> # ============================================================

>

> data_selected <- data_clean %>%

+ dplyr::select(cngrup, cnsex, h3tage, cnrandalt, h3isei, cngew,

+ Europe_vs_Others, cnerr, logMeth, e3part,

+ son5iq, son5kon, son5ver, son5mot, son5koo, son5mon)

>

> # Remove rows with any missing values

> data_complete <- na.omit(data_selected)

>

> cat("Original N:", nrow(data_selected), "\n")

Original N: 132

> cat("Complete cases N:", nrow(data_complete), "\n")

Complete cases N: 86

> cat("Rows removed:", nrow(data_selected) - nrow(data_complete), "\n")

Rows removed: 46

>

> # ============================================================

> # Step 3: Create z-scores for continuous variables

> # ============================================================

>

> data_complete <- data_complete %>%

+ mutate(

+ z_logMeth = as.numeric(scale(logMeth)),

+ z_h3isei = as.numeric(scale(h3isei)), # <--- CHANGED: was z_hisei

+ z_cngew = as.numeric(scale(cngew)),

+ z_h3tage = as.numeric(scale(h3tage)),

+ z_cnrandalt = as.numeric(scale(cnrandalt)),

+ z_son5iq = as.numeric(scale(son5iq)),

+ z_son5kon = as.numeric(scale(son5kon)),

+ z_son5ver = as.numeric(scale(son5ver)),

+ z_son5mot = as.numeric(scale(son5mot)),

+ z_son5koo = as.numeric(scale(son5koo)),

+ z_son5mon = as.numeric(scale(son5mon)),

+ z_e3part = as.numeric(scale(e3part))

+ )

>

> # Create reversed e3part (higher = more disagreement)

> data_complete$z_e3part_rev <- -data_complete$z_e3part

>

> cat("\nZ-scores created with h3isei.\n")

Z-scores

> cat("Final N for all models:", nrow(data_complete), "\n")

Final N for all models: 86

>

> # ============================================================

> # MODEL 1: z_logMeth -> z_son5iq

> # Covariates: cnsex, Europe_vs_Others, z_h3isei, z_cngew

> # ============================================================

>

> model1 <- lm(z_son5iq ~ z_logMeth + cnsex + Europe_vs_Others + z_h3isei + z_cngew,

+ data = data_complete)

>

> cat("\n========== MODEL 1 ==========\n")

========== MODEL 1 ==========

> summary(model1)

Call:

lm(formula = z_son5iq ~ z_logMeth + cnsex + Europe_vs_Others +

z_h3isei + z_cngew, data = data_complete)

Residuals:

Min 1Q Median 3Q Max

-2.28810 -0.58020 -0.04377 0.62783 1.95005

Coefficients:

Estimate Std. Error t value Pr(>|t|)

(Intercept) -0.1285 0.2238 -0.574 0.5673

z_logMeth -0.1100 0.1025 -1.073 0.2863

cnsex 0.4939 0.2096 2.357 0.0209 *

Europe_vs_Others -0.2274 0.2314 -0.983 0.3288

z_h3isei 0.2556 0.1045 2.446 0.0166 *

z_cngew 0.2297 0.1067 2.153 0.0344 *

---

Signif. codes: 0 ‘***’ 0.001 ‘**’ 0.01 ‘*’ 0.05 ‘.’ 0.1 ‘ ’ 1

Residual standard error: 0.9373 on 80 degrees of freedom

Multiple R-squared: 0.1731, Adjusted R-squared: 0.1214

F-statistic: 3.348 on 5 and 80 DF, p-value: 0.008496

>

> # ============================================================

> # MODEL 2: z_logMeth -> z_son5kon

> # Covariates: cnsex, Europe_vs_Others, z_h3isei, z_cngew, z_son5mon

> # ============================================================

>

> model2 <- lm(z_son5kon ~ z_logMeth + cnsex + Europe_vs_Others + z_h3isei + z_cngew + z_son5mon,

+ data = data_complete)

>

> cat("\n========== MODEL 2 ==========\n")

========== MODEL 2 ==========

> summary(model2)

Call:

lm(formula = z_son5kon ~ z_logMeth + cnsex + Europe_vs_Others +

z_h3isei + z_cngew + z_son5mon, data = data_complete)

Residuals:

Min 1Q Median 3Q Max

-1.4441 -0.7486 -0.1339 0.6262 2.6390

Coefficients:

Estimate Std. Error t value Pr(>|t|)

(Intercept) 0.13461 0.22671 0.594 0.55438

z_logMeth 0.31450 0.10258 3.066 0.00297 **

cnsex -0.45636 0.20987 -2.174 0.03266 *

Europe_vs_Others 0.18736 0.23598 0.794 0.42959

z_h3isei -0.08758 0.10521 -0.832 0.40764

z_cngew 0.11968 0.10951 1.093 0.27776

z_son5mon 0.11449 0.10561 1.084 0.28164

---

Signif. codes: 0 ‘***’ 0.001 ‘**’ 0.01 ‘*’ 0.05 ‘.’ 0.1 ‘ ’ 1

Residual standard error: 0.9383 on 79 degrees of freedom

Multiple R-squared: 0.1818, Adjusted R-squared: 0.1197

F-statistic: 2.926 on 6 and 79 DF, p-value: 0.01251

>

> # ============================================================

> # MODEL 3: z_logMeth -> z_son5ver, z_son5mot, z_son5koo

> # Covariates: cnsex, Europe_vs_Others, z_h3isei, z_cngew, z_son5mon

> # ============================================================

>

> model3_ver <- lm(z_son5ver ~ z_logMeth + cnsex + Europe_vs_Others + z_h3isei + z_cngew + z_son5mon,

+ data = data_complete)

> model3_mot <- lm(z_son5mot ~ z_logMeth + cnsex + Europe_vs_Others + z_h3isei + z_cngew + z_son5mon,

+ data = data_complete)

> model3_koo <- lm(z_son5koo ~ z_logMeth + cnsex + Europe_vs_Others + z_h3isei + z_cngew + z_son5mon,

+ data = data_complete)

>

> cat("\n========== MODEL 3a: z_logMeth -> z_son5ver ==========\n")

========== MODEL 3a: z_logMeth -> z_son5ver ==========

> summary(model3_ver)

Call:

lm(formula = z_son5ver ~ z_logMeth + cnsex + Europe_vs_Others +

z_h3isei + z_cngew + z_son5mon, data = data_complete)

Residuals:

Min 1Q Median 3Q Max

-1.3291 -0.4483 -0.2758 -0.0616 3.4007

Coefficients:

Estimate Std. Error t value Pr(>|t|)

(Intercept) 0.006657 0.238678 0.028 0.978

z_logMeth 0.203627 0.107996 1.886 0.063 .

cnsex -0.063299 0.220949 -0.286 0.775

Europe_vs_Others 0.043208 0.248431 0.174 0.862

z_h3isei -0.159480 0.110761 -1.440 0.154

z_cngew -0.084744 0.115287 -0.735 0.464

z_son5mon 0.144317 0.111187 1.298 0.198

---

Signif. codes: 0 ‘***’ 0.001 ‘**’ 0.01 ‘*’ 0.05 ‘.’ 0.1 ‘ ’ 1

Residual standard error: 0.9878 on 79 degrees of freedom

Multiple R-squared: 0.09317, Adjusted R-squared: 0.02429

F-statistic: 1.353 on 6 and 79 DF, p-value: 0.2441

> cat("\n========== MODEL 3b: z_logMeth -> z_son5mot ==========\n")

========== MODEL 3b: z_logMeth -> z_son5mot ==========

> summary(model3_mot)

Call:

lm(formula = z_son5mot ~ z_logMeth + cnsex + Europe_vs_Others +

z_h3isei + z_cngew + z_son5mon, data = data_complete)

Residuals:

Min 1Q Median 3Q Max

-1.0302 -0.6210 -0.4048 0.3821 3.3913

Coefficients:

Estimate Std. Error t value Pr(>|t|)

(Intercept) 0.17600 0.24114 0.730 0.4676

z_logMeth 0.05195 0.10911 0.476 0.6353

cnsex -0.06972 0.22323 -0.312 0.7556

Europe_vs_Others -0.19417 0.25100 -0.774 0.4415

z_h3isei -0.21169 0.11190 -1.892 0.0622 .

z_cngew 0.09326 0.11648 0.801 0.4257

z_son5mon -0.02769 0.11234 -0.246 0.8060

---

Signif. codes: 0 ‘***’ 0.001 ‘**’ 0.01 ‘*’ 0.05 ‘.’ 0.1 ‘ ’ 1

Residual standard error: 0.998 on 79 degrees of freedom

Multiple R-squared: 0.07434, Adjusted R-squared: 0.004042

F-statistic: 1.057 on 6 and 79 DF, p-value: 0.3952

> cat("\n========== MODEL 3c: z_logMeth -> z_son5koo ==========\n")

========== MODEL 3c: z_logMeth -> z_son5koo ==========

> summary(model3_koo)

Call:

lm(formula = z_son5koo ~ z_logMeth + cnsex + Europe_vs_Others +

z_h3isei + z_cngew + z_son5mon, data = data_complete)

Residuals:

Min 1Q Median 3Q Max

-1.0329 -0.5431 -0.3307 0.0773 3.8672

Coefficients:

Estimate Std. Error t value Pr(>|t|)

(Intercept) 0.21181 0.23871 0.887 0.3776

z_logMeth 0.01046 0.10801 0.097 0.9231

cnsex -0.25928 0.22098 -1.173 0.2442

Europe_vs_Others -0.08752 0.24847 -0.352 0.7256

z_h3isei -0.22652 0.11078 -2.045 0.0442 *

z_cngew 0.05802 0.11530 0.503 0.6162

z_son5mon 0.15556 0.11120 1.399 0.1658

---

Signif. codes: 0 ‘***’ 0.001 ‘**’ 0.01 ‘*’ 0.05 ‘.’ 0.1 ‘ ’ 1

Residual standard error: 0.9879 on 79 degrees of freedom

Multiple R-squared: 0.09289, Adjusted R-squared: 0.024

F-statistic: 1.348 on 6 and 79 DF, p-value: 0.246

>

> # ============================================================

> # MODEL 4: cngrup -> z_logMeth

> # Covariates: cnsex, Europe_vs_Others, z_h3isei, z_cngew, z_h3tage, z_cnrandalt, cnerr

> # ============================================================

>

> model4 <- lm(z_logMeth ~ cngrup + cnsex + Europe_vs_Others + z_h3isei + z_cngew +

+ z_h3tage + z_cnrandalt + cnerr,

+ data = data_complete)

>

> cat("\n========== MODEL 4 ==========\n")

========== MODEL 4 ==========

> summary(model4)

Call:

lm(formula = z_logMeth ~ cngrup + cnsex + Europe_vs_Others +

z_h3isei + z_cngew + z_h3tage + z_cnrandalt + cnerr, data = data_complete)

Residuals:

Min 1Q Median 3Q Max

-1.2036 -0.6074 -0.3106 0.2909 3.3237

Coefficients:

Estimate Std. Error t value Pr(>|t|)

(Intercept) -0.49476 0.35699 -1.386 0.1698

cngrup 0.01464 0.22864 0.064 0.9491

cnsex 0.06315 0.22113 0.286 0.7759

Europe_vs_Others 0.17464 0.24302 0.719 0.4745

z_h3isei -0.06957 0.11089 -0.627 0.5323

z_cngew 0.05322 0.11460 0.464 0.6437

z_h3tage -0.20989 0.10982 -1.911 0.0597 .

z_cnrandalt 0.29244 0.11194 2.612 0.0108 *

cnerr 0.38664 0.31441 1.230 0.2225

---

Signif. codes: 0 ‘***’ 0.001 ‘**’ 0.01 ‘*’ 0.05 ‘.’ 0.1 ‘ ’ 1

Residual standard error: 0.9764 on 77 degrees of freedom

Multiple R-squared: 0.1363, Adjusted R-squared: 0.04655

F-statistic: 1.519 on 8 and 77 DF, p-value: 0.1646

>

> # ============================================================

> # MODEL 5: Mediation cngrup -> z_logMeth -> z_son5kon

> # ============================================================

>

> med_model5 <- lm(z_logMeth ~ cngrup + cnerr + z_cnrandalt + z_h3tage +

+ cnsex + Europe_vs_Others + z_cngew + z_h3isei,

+ data = data_complete)

>

> out_model5 <- lm(z_son5kon ~ cngrup + z_logMeth + cnerr + z_cnrandalt +

+ cnsex + z_son5mon + Europe_vs_Others + z_h3isei + z_cngew,

+ data = data_complete)

>

> set.seed(2026)

> med5 <- mediate(med_model5, out_model5,

+ treat = "cngrup",

+ mediator = "z_logMeth",

+ sims = 1000,

+ boot = TRUE)

Running nonparametric bootstrap>

> cat("\n========== MODEL 5: cngrup -> z_logMeth -> z_son5kon ==========\n")

========== MODEL 5: cngrup -> z_logMeth -> z_son5kon ==========

> summary(med5)

Causal Mediation Analysis

Nonparametric Bootstrap Confidence Intervals with the Percentile Method

Estimate 95% CI Lower 95% CI Upper p-value

ACME 0.0038554 -0.1422266 0.1615643 0.876

ADE 0.0382444 -0.3554379 0.4118113 0.806

Total Effect 0.0420998 -0.3516585 0.4534384 0.808

Prop. Mediated 0.0915783 -4.6580921 6.1462935 0.708

Sample Size Used: 86

Simulations: 1000

>

> # ============================================================

> # MODEL 6: Mediation z_logMeth -> z_son5kon -> z_son5iq

> # ============================================================

>

> med_model6 <- lm(z_son5kon ~ z_logMeth + z_h3tage + cnsex +

+ Europe_vs_Others + z_cngew + z_h3isei + z_son5mon,

+ data = data_complete)

>

> out_model6 <- lm(z_son5iq ~ z_logMeth + z_son5kon + cnsex +

+ Europe_vs_Others + z_h3isei + z_cngew,

+ data = data_complete)

>

> set.seed(2026)

> med6 <- mediate(med_model6, out_model6,

+ treat = "z_logMeth",

+ mediator = "z_son5kon",

+ sims = 1000,

+ boot = TRUE)

Running nonparametric bootstrap>

> cat("\n========== MODEL 6: z_logMeth -> z_son5kon -> z_son5iq ==========\n")

========== MODEL 6: z_logMeth -> z_son5kon -> z_son5iq ==========

> summary(med6)

Causal Mediation Analysis

Nonparametric Bootstrap Confidence Intervals with the Percentile Method

Estimate 95% CI Lower 95% CI Upper p-value

ACME -0.0986658 -0.2215459 -0.0187365 0.012 *

ADE 0.0037977 -0.1647921 0.2478016 0.890

Total Effect -0.0948680 -0.2646672 0.1376257 0.396

Prop. Mediated 1.0400317 -13.7935244 9.3821351 0.400

---

Signif. codes: 0 ‘***’ 0.001 ‘**’ 0.01 ‘*’ 0.05 ‘.’ 0.1 ‘ ’ 1

Sample Size Used: 86

Simulations: 1000

>

> # ============================================================

> # MODEL 7: Mediation z_e3part_rev -> z_logMeth -> z_son5kon

> # ============================================================

>

> med_model7 <- lm(z_logMeth ~ z_e3part_rev + z_h3tage + cnsex +

+ Europe_vs_Others + z_cngew + z_h3isei,

+ data = data_complete)

>

> out_model7 <- lm(z_son5kon ~ z_e3part_rev + z_logMeth + cnsex +

+ z_son5mon + Europe_vs_Others + z_h3isei + z_cngew,

+ data = data_complete)

>

> set.seed(2026)

> med7 <- mediate(med_model7, out_model7,

+ treat = "z_e3part_rev",

+ mediator = "z_logMeth",

+ sims = 1000,

+ boot = TRUE)

Running nonparametric bootstrap>

> cat("\n========== MODEL 7: z_e3part_rev -> z_logMeth -> z_son5kon ==========\n")

========== MODEL 7: z_e3part_rev -> z_logMeth -> z_son5kon ==========

> summary(med7)

Causal Mediation Analysis

Nonparametric Bootstrap Confidence Intervals with the Percentile Method

Estimate 95% CI Lower 95% CI Upper p-value

ACME 0.0759067 -0.0065032 0.2154302 0.076 .

ADE -0.0142656 -0.2251060 0.1583064 0.878

Total Effect 0.0616411 -0.1426862 0.2515811 0.492

Prop. Mediated 1.2314307 -7.0321355 10.1674359 0.468

---

Signif. codes: 0 ‘***’ 0.001 ‘**’ 0.01 ‘*’ 0.05 ‘.’ 0.1 ‘ ’ 1

Sample Size Used: 86

Simulations: 1000

>

> # ============================================================

> # MODEL 8: Moderated Mediation (separate groups)

> # ============================================================

>

> # Control Group (cngrup = 0)

> cg_data <- data_complete[data_complete$cngrup == 0, ]

> ig_data <- data_complete[data_complete$cngrup == 1, ]

>

> cat("\n========== MODEL 8: Moderated Mediation ==========\n")

========== MODEL 8: Moderated Mediation ==========

> cat("Control Group N:", nrow(cg_data), "\n")

Control Group N: 39

> cat("Intervention Group N:", nrow(ig_data), "\n")

Intervention Group N: 47

>

> # Control Group mediation

> if(nrow(cg_data) >= 20) {

+ med_cg <- lm(z_logMeth ~ z_e3part_rev + z_h3tage + cnsex +

+ Europe_vs_Others + z_cngew + z_h3isei,

+ data = cg_data)

+ out_cg <- lm(z_son5kon ~ z_e3part_rev + z_logMeth + cnsex +

+ z_son5mon + Europe_vs_Others + z_h3isei + z_cngew,

+ data = cg_data)

+

+ set.seed(2026)

+ med8_cg <- mediate(med_cg, out_cg,

+ treat = "z_e3part_rev",

+ mediator = "z_logMeth",

+ sims = 1000,

+ boot = TRUE)

+ cat("\nControl Group:\n")

+ print(summary(med8_cg))

+ }

Running nonparametric bootstrap

Control Group:

Causal Mediation Analysis

Nonparametric Bootstrap Confidence Intervals with the Percentile Method

Estimate 95% CI Lower 95% CI Upper p-value

ACME 0.127805 -0.054882 0.422445 0.358

ADE -0.068779 -0.604624 0.388801 0.692

Total Effect 0.059026 -0.529532 0.503156 0.856

Prop. Mediated 2.165240 -5.532001 6.843259 0.678

Sample Size Used: 39

Simulations: 1000

>

> # Intervention Group mediation

> if(nrow(ig_data) >= 20) {

+ med_ig <- lm(z_logMeth ~ z_e3part_rev + z_h3tage + cnsex +

+ Europe_vs_Others + z_cngew + z_h3isei,

+ data = ig_data)

+ out_ig <- lm(z_son5kon ~ z_e3part_rev + z_logMeth + cnsex +

+ z_son5mon + Europe_vs_Others + z_h3isei + z_cngew,

+ data = ig_data)

+

+ set.seed(2026)

+ med8_ig <- mediate(med_ig, out_ig,

+ treat = "z_e3part_rev",

+ mediator = "z_logMeth",

+ sims = 1000,

+ boot = TRUE)

+ cat("\nIntervention Group:\n")

+ print(summary(med8_ig))

+ }

Running nonparametric bootstrap

Intervention Group:

Causal Mediation Analysis

Nonparametric Bootstrap Confidence Intervals with the Percentile Method

Estimate 95% CI Lower 95% CI Upper p-value

ACME 0.054892 -0.065009 0.196724 0.364

ADE -0.021212 -0.279439 0.210536 0.998

Total Effect 0.033681 -0.186221 0.259134 0.720

Prop. Mediated 1.629791 -6.973615 7.920586 0.788

Sample Size Used: 47

Simulations: 1000

>

> # ============================================================

> # MODEL 9: Serial Mediation

> # z_e3part_rev -> z_logMeth -> z_son5kon -> z_son5iq

> # ============================================================

>

> cat("\n========== MODEL 9: Serial Mediation ==========\n")

========== MODEL 9: Serial Mediation ==========

>

> # Path a: z_e3part_rev -> z_logMeth

> model_a <- lm(z_logMeth ~ z_e3part_rev + cnsex + z_h3tage +

+ Europe_vs_Others + z_cngew + z_h3isei,

+ data = data_complete)

> a_coef <- coef(model_a)["z_e3part_rev"]

>

> # Path b1: z_logMeth -> z_son5kon

> model_b1 <- lm(z_son5kon ~ z_logMeth + cnsex + z_son5mon +

+ Europe_vs_Others + z_cngew + z_h3isei,

+ data = data_complete)

> b1_coef <- coef(model_b1)["z_logMeth"]

>

> # Path b2: z_son5kon -> z_son5iq

> model_b2 <- lm(z_son5iq ~ z_son5kon + cnsex + Europe_vs_Others + z_cngew + z_h3isei,

+ data = data_complete)

> b2_coef <- coef(model_b2)["z_son5kon"]

>

> # Indirect effects

> ab1 <- a_coef * b1_coef

> ab2 <- b1_coef * b2_coef

> ab_serial <- a_coef * b1_coef * b2_coef

> total_indirect <- ab1 + ab2 + ab_serial

>

> # Total effect

> model_total <- lm(z_son5iq ~ z_e3part_rev + cnsex + Europe_vs_Others + z_cngew + z_h3isei,

+ data = data_complete)

> total_effect <- coef(model_total)["z_e3part_rev"]

>

> # Direct effect (c')

> model_cprime <- lm(z_son5iq ~ z_e3part_rev + z_logMeth + z_son5kon +

+ cnsex + Europe_vs_Others + z_cngew + z_h3isei,

+ data = data_complete)

> cprime_coef <- coef(model_cprime)["z_e3part_rev"]

>

> # Bootstrap for serial indirect effect

> boot_serial <- function(data, indices) {

+ d <- data[indices, ]

+ a_boot <- coef(lm(z_logMeth ~ z_e3part_rev + cnsex + z_h3tage +

+ Europe_vs_Others + z_cngew + z_h3isei, data = d))["z_e3part_rev"]

+ b1_boot <- coef(lm(z_son5kon ~ z_logMeth + cnsex + z_son5mon +

+ Europe_vs_Others + z_cngew + z_h3isei, data = d))["z_logMeth"]

+ b2_boot <- coef(lm(z_son5iq ~ z_son5kon + cnsex + Europe_vs_Others +

+ z_cngew + z_h3isei, data = d))["z_son5kon"]

+ return(a_boot * b1_boot * b2_boot)

+ }

>

> set.seed(2026)

> boot_result <- boot::boot(data_complete, boot_serial, R = 1000)

> boot_se <- sd(boot_result$t, na.rm = TRUE)

> boot_ci <- quantile(boot_result$t, c(0.025, 0.975), na.rm = TRUE)

> p_serial <- 2 * (1 - pnorm(abs(ab_serial / boot_se)))

>

> cat("\nSerial Mediation Results:\n")

Serial Mediation Results:

> cat(" a (z_e3part_rev -> z_logMeth):", round(a_coef, 4), "\n")

a (z_e3part_rev -> z_logMeth): 0.2388

> cat(" b1 (z_logMeth -> z_son5kon):", round(b1_coef, 4), "\n")

b1 (z_logMeth -> z_son5kon): 0.3145

> cat(" b2 (z_son5kon -> z_son5iq):", round(b2_coef, 4), "\n")

b2 (z_son5kon -> z_son5iq): -0.3605

> cat(" Indirect via methylation only (ab1):", round(ab1, 4), "\n")

Indirect via methylation only (ab1): 0.0751

> cat(" Indirect via concentration only (ab2):", round(ab2, 4), "\n")

Indirect via concentration only (ab2): -0.1134

> cat(" Serial indirect (a*b1*b2):", round(ab_serial, 4), "\n")

Serial indirect (a*b1*b2): -0.0271

> cat(" Boot SE:", round(boot_se, 4), "\n")

Boot SE: 0.0234

> cat(" Boot 95% CI: [", round(boot_ci[1], 4), ",", round(boot_ci[2], 4), "]\n")

Boot 95% CI: [ -0.0856 , 0.0023 ]

> cat(" p-value:", round(p_serial, 4), "\n")

p-value: 0.2477

> cat(" Total indirect:", round(total_indirect, 4), "\n")

Total indirect: -0.0654

> cat(" Direct effect (c'):", round(cprime_coef, 4), "\n")

Direct effect (c'): -0.0287

> cat(" Total effect:", round(total_effect, 4), "\n")

Total effect: -0.045

>

> # ============================================================

> # MODEL 10: Moderated Serial Mediation (IG vs CG)

> # ============================================================

>

> cat("\n========== MODEL 10: Moderated Serial Mediation ==========\n")

========== MODEL 10: Moderated Serial Mediation ==========

>

> run_serial_group <- function(data, group_name) {

+

+ cat("\n", group_name, "N =", nrow(data), "\n")

+

+ if(nrow(data) < 20) {

+ return(list(ab_serial = NA, p = NA, n = nrow(data)))

+ }

+

+ # Paths

+ model_a <- lm(z_logMeth ~ z_e3part_rev + cnsex + z_h3tage +

+ Europe_vs_Others + z_cngew + z_h3isei, data = data)

+ a_coef <- coef(model_a)["z_e3part_rev"]

+

+ model_b1 <- lm(z_son5kon ~ z_logMeth + cnsex + z_son5mon +

+ Europe_vs_Others + z_cngew + z_h3isei, data = data)

+ b1_coef <- coef(model_b1)["z_logMeth"]

+

+ model_b2 <- lm(z_son5iq ~ z_son5kon + cnsex + Europe_vs_Others + z_cngew + z_h3isei,

+ data = data)

+ b2_coef <- coef(model_b2)["z_son5kon"]

+

+ ab_serial <- a_coef * b1_coef * b2_coef

+

+ # Bootstrap

+ boot_serial <- function(d, indices) {

+ d_sub <- d[indices, ]

+ a_boot <- coef(lm(z_logMeth ~ z_e3part_rev + cnsex + z_h3tage +

+ Europe_vs_Others + z_cngew + z_h3isei, data = d_sub))["z_e3part_rev"]

+ b1_boot <- coef(lm(z_son5kon ~ z_logMeth + cnsex + z_son5mon +

+ Europe_vs_Others + z_cngew + z_h3isei, data = d_sub))["z_logMeth"]

+ b2_boot <- coef(lm(z_son5iq ~ z_son5kon + cnsex + Europe_vs_Others +

+ z_cngew + z_h3isei, data = d_sub))["z_son5kon"]

+ return(a_boot * b1_boot * b2_boot)

+ }

+

+ set.seed(2026)

+ boot_result <- boot::boot(data, boot_serial, R = 500)

+ boot_se <- sd(boot_result$t, na.rm = TRUE)

+ p_serial <- 2 * (1 - pnorm(abs(ab_serial / boot_se)))

+

+ return(list(ab_serial = ab_serial, p = p_serial, n = nrow(data),

+ a = a_coef, b1 = b1_coef, b2 = b2_coef))

+ }

>

> # Run for both groups

> cg_serial <- run_serial_group(data_complete[data_complete$cngrup == 0, ], "Control Group")

Control Group N = 39

> ig_serial <- run_serial_group(data_complete[data_complete$cngrup == 1, ], "Intervention Group")

Intervention Group N = 47

>

> cat("\n--- Results ---\n")

--- Results ---

> cat("Control Group:\n")

Control Group:

> cat(" N:", cg_serial$n, "\n")

N: 39

> cat(" Serial indirect:", round(cg_serial$ab_serial, 4), "\n")

Serial indirect: -0.0555

> cat(" p-value:", round(cg_serial$p, 4), "\n\n")

p-value: 0.384

>

> cat("Intervention Group:\n")

Intervention Group:

> cat(" N:", ig_serial$n, "\n")

N: 47

> cat(" Serial indirect:", round(ig_serial$ab_serial, 4), "\n")

Serial indirect: -0.018

> cat(" p-value:", round(ig_serial$p, 4), "\n\n")

p-value: 0.3736

>

> # Test difference

> if(!is.na(cg_serial$ab_serial) & !is.na(ig_serial$ab_serial)) {

+ diff_serial <- ig_serial$ab_serial - cg_serial$ab_serial

+ cat("Difference (IG - CG):", round(diff_serial, 4), "\n")

+ }

Difference (IG - CG): 0.0375

**NMAR analyses**

> library(dplyr)

> library(mediation)

> library(boot)

> # Load raw data

> raw_data <- read.csv("original dataset.csv")

> dummy_codes <- c(-333, -444, -555, -777, -999)

> data_clean <- raw_data

> for(code in dummy_codes) {

+ data_clean[data_clean == code] <- NA

+ }

> data_selected <- data_clean %>%

+ dplyr::select(cngrup, cnsex, h3tage, cnrandalt, h3isei, cngew,

+ Europe_vs_Others, cnerr, logMeth, e3part,

+ son5iq, son5kon, son5ver, son5mot, son5koo, son5mon)

> missing_h3isei <- is.na(data_selected$h3isei)

> cat("Missing on h3isei:", sum(missing_h3isei),

+ "(", round(mean(missing_h3isei)*100, 1), "%)\n")

Missing on h3isei: 16 ( 12.1 %)

> set.seed(2026)

> observed_h3isei <- data_selected$h3isei[!missing_h3isei]

> mean_h3isei <- mean(observed_h3isei, na.rm = TRUE)

> sd_h3isei <- sd(observed_h3isei, na.rm = TRUE)

> cat("\nObserved h3isei - Mean:", round(mean_h3isei, 3),

+ "SD:", round(sd_h3isei, 3), "\n")

Observed h3isei - Mean: 34.44 SD: 23.206

> # Shift -1 SD (lower values for missing)

> shift_h3isei <- -1

> imputed_h3isei <- mean_h3isei + shift_h3isei * sd_h3isei +

+ rnorm(sum(missing_h3isei), 0, sd_h3isei * 0.3)

> cat("Imputed h3isei (shift -1 SD) - Mean:", round(mean(imputed_h3isei), 3),

+ "SD:", round(sd(imputed_h3isei), 3), "\n")

Imputed h3isei (shift -1 SD) - Mean: 7.497 SD: 6.856

> data_mnar <- data_selected

> # Impute h3isei (shifted down)

> data_mnar$h3isei[missing_h3isei] <- imputed_h3isei

> # For other variables, use median imputation (MAR)

> impute_with_median <- function(x) {

+ ifelse(is.na(x), median(x, na.rm = TRUE), x)

+ }

> numeric_vars <- sapply(data_mnar, is.numeric)

> data_mnar[, numeric_vars] <- lapply(data_mnar[, numeric_vars], impute_with_median)

> # Remove any remaining NA

> data_complete <- na.omit(data_mnar)

> cat("\nFinal MNAR dataset N (Models 1-6):", nrow(data_complete), "\n")

Final MNAR dataset N (Models 1-6): 132

> cat("Shift applied: h3isei -1 SD\n")

Shift applied: h3isei -1 SD

> data_complete <- data_complete %>%

+ mutate(

+ z_logMeth = as.numeric(scale(logMeth)),

+ z_h3isei = as.numeric(scale(h3isei)),

+ z_cngew = as.numeric(scale(cngew)),

+ z_h3tage = as.numeric(scale(h3tage)),

+ z_cnrandalt = as.numeric(scale(cnrandalt)),

+ z_son5iq = as.numeric(scale(son5iq)),

+ z_son5kon = as.numeric(scale(son5kon)),

+ z_son5ver = as.numeric(scale(son5ver)),

+ z_son5mot = as.numeric(scale(son5mot)),

+ z_son5koo = as.numeric(scale(son5koo)),

+ z_son5mon = as.numeric(scale(son5mon)),

+ z_e3part = as.numeric(scale(e3part))

+ )

> cat("\nZ-scores created.\n")

Z-scores created.

> model1 <- lm(z_son5iq ~ z_logMeth + cnsex + Europe_vs_Others + z_h3isei + z_cngew,

+ data = data_complete)

> cat("\n========== MODEL 1 (MNAR, h3isei -1 SD) ==========\n")

========== MODEL 1 (MNAR, h3isei -1 SD) ==========

> summary(model1)

Call:

lm(formula = z_son5iq ~ z_logMeth + cnsex + Europe_vs_Others +

z_h3isei + z_cngew, data = data_complete)

Residuals:

Min 1Q Median 3Q Max

-2.47479 -0.50615 -0.00185 0.59697 2.18711

Coefficients:

Estimate Std. Error t value Pr(>|t|)

(Intercept) -0.08975 0.18431 -0.487 0.62715

z_logMeth -0.07957 0.08406 -0.947 0.34569

cnsex 0.30212 0.16897 1.788 0.07619 .

Europe_vs_Others -0.10538 0.19225 -0.548 0.58454

z_h3isei 0.23026 0.08625 2.670 0.00859 **

z_cngew 0.19330 0.08583 2.252 0.02604 *

---

Signif. codes: 0 ‘***’ 0.001 ‘**’ 0.01 ‘*’ 0.05 ‘.’ 0.1 ‘ ’ 1

Residual standard error: 0.9594 on 126 degrees of freedom

Multiple R-squared: 0.1147, Adjusted R-squared: 0.07953

F-statistic: 3.264 on 5 and 126 DF, p-value: 0.008339

> model2 <- lm(z_son5kon ~ z_logMeth + cnsex + Europe_vs_Others + z_h3isei + z_cngew + z_son5mon,

+ data = data_complete)

> cat("\n========== MODEL 2 (MNAR, h3isei -1 SD) ==========\n")

========== MODEL 2 (MNAR, h3isei -1 SD) ==========

> summary(model2)

Call:

lm(formula = z_son5kon ~ z_logMeth + cnsex + Europe_vs_Others +

z_h3isei + z_cngew + z_son5mon, data = data_complete)

Residuals:

Min 1Q Median 3Q Max

-1.5858 -0.8407 0.1452 0.5663 3.1910

Coefficients:

Estimate Std. Error t value Pr(>|t|)

(Intercept) 0.191795 0.190135 1.009 0.31505

z_logMeth 0.225264 0.085357 2.639 0.00937 **

cnsex -0.377073 0.173897 -2.168 0.03202 *

Europe_vs_Others 0.019493 0.196807 0.099 0.92126

z_h3isei -0.122945 0.087795 -1.400 0.16388

z_cngew 0.028394 0.088760 0.320 0.74958

z_son5mon -0.003638 0.088195 -0.041 0.96716

---

Signif. codes: 0 ‘***’ 0.001 ‘**’ 0.01 ‘*’ 0.05 ‘.’ 0.1 ‘ ’ 1

Residual standard error: 0.9737 on 125 degrees of freedom

Multiple R-squared: 0.09531, Adjusted R-squared: 0.05189

F-statistic: 2.195 on 6 and 125 DF, p-value: 0.04774

> model3_ver <- lm(z_son5ver ~ z_logMeth + cnsex + Europe_vs_Others + z_h3isei + z_cngew + z_son5mon,

+ data = data_complete)

> model3_mot <- lm(z_son5mot ~ z_logMeth + cnsex + Europe_vs_Others + z_h3isei + z_cngew + z_son5mon,

+ data = data_complete)

> model3_koo <- lm(z_son5koo ~ z_logMeth + cnsex + Europe_vs_Others + z_h3isei + z_cngew + z_son5mon,

+ data = data_complete)

> cat("\n========== MODEL 3a (MNAR): z_logMeth -> z_son5ver ==========\n")

========== MODEL 3a (MNAR): z_logMeth -> z_son5ver ==========

> summary(model3_ver)

Call:

lm(formula = z_son5ver ~ z_logMeth + cnsex + Europe_vs_Others +

z_h3isei + z_cngew + z_son5mon, data = data_complete)

Residuals:

Min 1Q Median 3Q Max

-1.1951 -0.3914 -0.2643 -0.0912 3.9511

Coefficients:

Estimate Std. Error t value Pr(>|t|)

(Intercept) 0.09293 0.19371 0.480 0.6323

z_logMeth 0.18296 0.08696 2.104 0.0374 *

cnsex -0.07568 0.17717 -0.427 0.6700

Europe_vs_Others -0.07253 0.20051 -0.362 0.7182

z_h3isei -0.11968 0.08945 -1.338 0.1833

z_cngew -0.07303 0.09043 -0.808 0.4209

z_son5mon 0.07478 0.08985 0.832 0.4068

---

Signif. codes: 0 ‘***’ 0.001 ‘**’ 0.01 ‘*’ 0.05 ‘.’ 0.1 ‘ ’ 1

Residual standard error: 0.992 on 125 degrees of freedom

Multiple R-squared: 0.06097, Adjusted R-squared: 0.01589

F-statistic: 1.353 on 6 and 125 DF, p-value: 0.239

> cat("\n========== MODEL 3b (MNAR): z_logMeth -> z_son5mot ==========\n")

========== MODEL 3b (MNAR): z_logMeth -> z_son5mot ==========

> summary(model3_mot)

Call:

lm(formula = z_son5mot ~ z_logMeth + cnsex + Europe_vs_Others +

z_h3isei + z_cngew + z_son5mon, data = data_complete)

Residuals:

Min 1Q Median 3Q Max

-0.9581 -0.6741 -0.4114 0.5872 3.5299

Coefficients:

Estimate Std. Error t value Pr(>|t|)

(Intercept) 0.20762 0.19319 1.075 0.2846

z_logMeth 0.06863 0.08673 0.791 0.4303

cnsex -0.11072 0.17670 -0.627 0.5321

Europe_vs_Others -0.20675 0.19997 -1.034 0.3032

z_h3isei -0.20561 0.08921 -2.305 0.0228 *

z_cngew -0.03758 0.09019 -0.417 0.6776

z_son5mon -0.03429 0.08961 -0.383 0.7026

---

Signif. codes: 0 ‘***’ 0.001 ‘**’ 0.01 ‘*’ 0.05 ‘.’ 0.1 ‘ ’ 1

Residual standard error: 0.9894 on 125 degrees of freedom

Multiple R-squared: 0.06596, Adjusted R-squared: 0.02113

F-statistic: 1.471 on 6 and 125 DF, p-value: 0.1933

> cat("\n========== MODEL 3c (MNAR): z_logMeth -> z_son5koo ==========\n")

========== MODEL 3c (MNAR): z_logMeth -> z_son5koo ==========

> summary(model3_koo)

Call:

lm(formula = z_son5koo ~ z_logMeth + cnsex + Europe_vs_Others +

z_h3isei + z_cngew + z_son5mon, data = data_complete)

Residuals:

Min 1Q Median 3Q Max

-0.9346 -0.4605 -0.2973 -0.0904 4.7858

Coefficients:

Estimate Std. Error t value Pr(>|t|)

(Intercept) 0.2784702 0.1944822 1.432 0.155

z_logMeth 0.0093324 0.0873082 0.107 0.915

cnsex -0.1366084 0.1778733 -0.768 0.444

Europe_vs_Others -0.2864070 0.2013073 -1.423 0.157

z_h3isei -0.1394891 0.0898022 -1.553 0.123

z_cngew 0.0002158 0.0907898 0.002 0.998

z_son5mon 0.0558172 0.0902111 0.619 0.537

Residual standard error: 0.996 on 125 degrees of freedom

Multiple R-squared: 0.05347, Adjusted R-squared: 0.008034

F-statistic: 1.177 on 6 and 125 DF, p-value: 0.3229

> model4 <- lm(z_logMeth ~ cngrup + cnsex + Europe_vs_Others + z_h3isei + z_cngew +

+ z_h3tage + z_cnrandalt + cnerr,

+ data = data_complete)

> cat("\n========== MODEL 4 (MNAR, h3isei -1 SD) ==========\n")

========== MODEL 4 (MNAR, h3isei -1 SD) ==========

> summary(model4)

Call:

lm(formula = z_logMeth ~ cngrup + cnsex + Europe_vs_Others +

z_h3isei + z_cngew + z_h3tage + z_cnrandalt + cnerr, data = data_complete)

Residuals:

Min 1Q Median 3Q Max

-1.2317 -0.6131 -0.2772 0.2390 3.6965

Coefficients:

Estimate Std. Error t value Pr(>|t|)

(Intercept) -0.33216 0.30616 -1.085 0.28007

cngrup -0.36692 0.17958 -2.043 0.04317 *

cnsex 0.15506 0.17313 0.896 0.37221

Europe_vs_Others 0.06878 0.20022 0.344 0.73178

z_h3isei -0.04598 0.08952 -0.514 0.60839

z_cngew -0.04346 0.08861 -0.490 0.62467

z_h3tage -0.13840 0.08909 -1.553 0.12288

z_cnrandalt 0.23504 0.08951 2.626 0.00974 **

cnerr 0.45374 0.26831 1.691 0.09335 .

---

Signif. codes: 0 ‘***’ 0.001 ‘**’ 0.01 ‘*’ 0.05 ‘.’ 0.1 ‘ ’ 1

Residual standard error: 0.9745 on 123 degrees of freedom

Multiple R-squared: 0.1084, Adjusted R-squared: 0.05043

F-statistic: 1.87 on 8 and 123 DF, p-value: 0.07067

> med_model5 <- lm(z_logMeth ~ cngrup + cnerr + z_cnrandalt + z_h3tage +

+ cnsex + Europe_vs_Others + z_cngew + z_h3isei,

+ data = data_complete)

> out_model5 <- lm(z_son5kon ~ cngrup + z_logMeth + cnerr + z_cnrandalt +

+ cnsex + z_son5mon + Europe_vs_Others + z_h3isei + z_cngew,

+ data = data_complete)

> set.seed(2026)

> med5 <- mediate(med_model5, out_model5,

+ treat = "cngrup",

+ mediator = "z_logMeth",

+ sims = 1000,

+ boot = TRUE)

Running nonparametric bootstrap> cat("\n========== MODEL 5 (MNAR): cngrup -> z_logMeth -> z_son5kon ==========\n")

========== MODEL 5 (MNAR): cngrup -> z_logMeth -> z_son5kon ==========

> summary(med5)

Causal Mediation Analysis

Nonparametric Bootstrap Confidence Intervals with the Percentile Method

Estimate 95% CI Lower 95% CI Upper p-value

ACME -0.076318 -0.190301 0.011892 0.110

ADE 0.028458 -0.373995 0.376441 0.864

Total Effect -0.047861 -0.439014 0.314359 0.806

Prop. Mediated 1.594594 -7.533051 6.723051 0.820

Sample Size Used: 132

Simulations: 1000

> med_model6 <- lm(z_son5kon ~ z_logMeth + z_h3tage + cnsex +

+ Europe_vs_Others + z_cngew + z_h3isei + z_son5mon,

+ data = data_complete)

> out_model6 <- lm(z_son5iq ~ z_logMeth + z_son5kon + cnsex +

+ Europe_vs_Others + z_h3isei + z_cngew,

+ data = data_complete)

> set.seed(2026)

> med6 <- mediate(med_model6, out_model6,

+ treat = "z_logMeth",

+ mediator = "z_son5kon",

+ sims = 1000,

+ boot = TRUE)

Running nonparametric bootstrap> cat("\n========== MODEL 6 (MNAR): z_logMeth -> z_son5kon -> z_son5iq ==========\n")

========== MODEL 6 (MNAR): z_logMeth -> z_son5kon -> z_son5iq ==========

> summary(med6)

Causal Mediation Analysis

Nonparametric Bootstrap Confidence Intervals with the Percentile Method

Estimate 95% CI Lower 95% CI Upper p-value

ACME -0.0780908 -0.1561031 -0.0096382 0.032 *

ADE 0.0029809 -0.1451244 0.1926256 0.932

Total Effect -0.0751098 -0.2275144 0.1129509 0.430

Prop. Mediated 1.0396878 -10.9486968 8.5211390 0.426

---

Signif. codes: 0 ‘***’ 0.001 ‘**’ 0.01 ‘*’ 0.05 ‘.’ 0.1 ‘ ’ 1

Sample Size Used: 132

Simulations: 1000

> med_model7 <- lm(z_logMeth ~ z_e3part_rev + z_h3tage + cnsex +

+ Europe_vs_Others + z_cngew + z_h3isei,

+ data = data_complete)

> out_model7 <- lm(z_son5kon ~ z_e3part_rev + z_logMeth + cnsex +

+ z_son5mon + Europe_vs_Others + z_h3isei + z_cngew,

+ data = data_complete)

> set.seed(2026)

> med7 <- mediate(med_model7, out_model7,

+ treat = "z_e3part_rev",

+ mediator = "z_logMeth",

+ sims = 1000,

+ boot = TRUE)

Running nonparametric bootstrap> cat("\n========== MODEL 7 (MNAR) ==========\n")

========== MODEL 7 (MNAR) ==========

> cat("z_e3part_rev -> z_logMeth -> z_son5kon\n")

z_e3part_rev -> z_logMeth -> z_son5kon

> cat("Shift: e3part_rev +1 SD, h3isei -1 SD\n")

Shift: e3part_rev +1 SD, h3isei -1 SD

> summary(med7)

Causal Mediation Analysis

Nonparametric Bootstrap Confidence Intervals with the Percentile Method

Estimate 95% CI Lower 95% CI Upper p-value

ACME 0.0536573 0.0028909 0.1293542 0.032 *

ADE 0.0260346 -0.1675971 0.2008616 0.792

Total Effect 0.0796919 -0.1034261 0.2489432 0.370

Prop. Mediated 0.6733093 -4.1153945 6.8675909 0.374

---

Signif. codes: 0 ‘***’ 0.001 ‘**’ 0.01 ‘*’ 0.05 ‘.’ 0.1 ‘ ’ 1

Sample Size Used: 132

Simulations: 1000

> cg_data <- data_complete[data_complete$cngrup == 0, ]

> ig_data <- data_complete[data_complete$cngrup == 1, ]

> cat("\n========== MODEL 8 (MNAR) ==========\n")

========== MODEL 8 (MNAR) ==========

> cat("Control Group N:", nrow(cg_data), "\n")

Control Group N: 60

> cat("Intervention Group N:", nrow(ig_data), "\n")

Intervention Group N: 72

> if(nrow(cg_data) >= 20) {

+ med_cg <- lm(z_logMeth ~ z_e3part_rev + z_h3tage + cnsex +

+ Europe_vs_Others + z_cngew + z_h3isei,

+ data = cg_data)

+ out_cg <- lm(z_son5kon ~ z_e3part_rev + z_logMeth + cnsex +

+ z_son5mon + Europe_vs_Others + z_h3isei + z_cngew,

+ data = cg_data)

+

+ set.seed(2026)

+ med8_cg <- mediate(med_cg, out_cg,

+ treat = "z_e3part_rev",

+ mediator = "z_logMeth",

+ sims = 1000,

+ boot = TRUE)

+ cat("\nControl Group:\n")

+ print(summary(med8_cg))

+ }

Running nonparametric bootstrap

Control Group:

Causal Mediation Analysis

Nonparametric Bootstrap Confidence Intervals with the Percentile Method

Estimate 95% CI Lower 95% CI Upper p-value

ACME 0.067069 -0.058259 0.273373 0.292

ADE 0.081263 -0.298525 0.368472 0.646

Total Effect 0.148331 -0.210913 0.450828 0.336

Prop. Mediated 0.452156 -3.666973 4.525034 0.492

Sample Size Used: 60

Simulations: 1000

> if(nrow(ig_data) >= 20) {

+ med_ig <- lm(z_logMeth ~ z_e3part_rev + z_h3tage + cnsex +

+ Europe_vs_Others + z_cngew + z_h3isei,

+ data = ig_data)

+ out_ig <- lm(z_son5kon ~ z_e3part_rev + z_logMeth + cnsex +

+ z_son5mon + Europe_vs_Others + z_h3isei + z_cngew,

+ data = ig_data)

+

+ set.seed(2026)

+ med8_ig <- mediate(med_ig, out_ig,

+ treat = "z_e3part_rev",

+ mediator = "z_logMeth",

+ sims = 1000,

+ boot = TRUE)

+ cat("\nIntervention Group:\n")

+ print(summary(med8_ig))

+ }

Running nonparametric bootstrap

Intervention Group:

Causal Mediation Analysis

Nonparametric Bootstrap Confidence Intervals with the Percentile Method

Estimate 95% CI Lower 95% CI Upper p-value

ACME 0.0289918 -0.0401671 0.1089654 0.360

ADE 0.0038628 -0.2392135 0.2205546 0.882

Total Effect 0.0328546 -0.1839784 0.2458732 0.758

Prop. Mediated 0.8824273 -5.3588417 4.0893249 0.830

Sample Size Used: 72

Simulations: 1000

> cat("\n========== MODEL 9 (MNAR) ==========\n")

========== MODEL 9 (MNAR) ==========

> model_a <- lm(z_logMeth ~ z_e3part_rev + cnsex + z_h3tage +

+ Europe_vs_Others + z_cngew + z_h3isei,

+ data = data_complete)

> a_coef <- coef(model_a)["z_e3part_rev"]

> model_b1 <- lm(z_son5kon ~ z_logMeth + cnsex + z_son5mon +

+ Europe_vs_Others + z_cngew + z_h3isei,

+ data = data_complete)

> b1_coef <- coef(model_b1)["z_logMeth"]

> model_b2 <- lm(z_son5iq ~ z_son5kon + cnsex + Europe_vs_Others + z_cngew + z_h3isei,

+ data = data_complete)

> b2_coef <- coef(model_b2)["z_son5kon"]

> ab_serial <- a_coef * b1_coef * b2_coef

> boot_serial <- function(data, indices) {

+ d <- data[indices, ]

+ a_boot <- coef(lm(z_logMeth ~ z_e3part_rev + cnsex + z_h3tage +

+ Europe_vs_Others + z_cngew + z_h3isei, data = d))["z_e3part_rev"]

+ b1_boot <- coef(lm(z_son5kon ~ z_logMeth + cnsex + z_son5mon +

+ Europe_vs_Others + z_cngew + z_h3isei, data = d))["z_logMeth"]

+ b2_boot <- coef(lm(z_son5iq ~ z_son5kon + cnsex + Europe_vs_Others +

+ z_cngew + z_h3isei, data = d))["z_son5kon"]

+ return(a_boot * b1_boot * b2_boot)

+ }

> set.seed(2026)

> boot_result <- boot::boot(data_complete, boot_serial, R = 1000)

> boot_se <- sd(boot_result$t, na.rm = TRUE)

> boot_ci <- quantile(boot_result$t, c(0.025, 0.975), na.rm = TRUE)

> p_serial <- 2 * (1 - pnorm(abs(ab_serial / boot_se)))

> cat("\nSerial Mediation Results (MNAR):\n")

Serial Mediation Results (MNAR):

> cat(" a:", round(a_coef, 4), "\n")

a: 0.2463

> cat(" b1:", round(b1_coef, 4), "\n")

b1: 0.2239

> cat(" b2:", round(b2_coef, 4), "\n")

b2: -0.3657

> cat(" Serial indirect (a*b1*b2):", round(ab_serial, 4), "\n")

Serial indirect (a*b1*b2): -0.0202

> cat(" Boot SE:", round(boot_se, 4), "\n")

Boot SE: 0.0139

> cat(" Boot 95% CI: [", round(boot_ci[1], 4), ",", round(boot_ci[2], 4), "]\n")

Boot 95% CI: [ -0.0529 , -0.0013 ]

> cat(" p-value:", round(p_serial, 4), "\n")

p-value: 0.1462

> cat("\n========== MODEL 10 (MNAR) ==========\n")

========== MODEL 10 (MNAR) ==========

> run_serial_group <- function(data, group_name) {

+

+ cat("\n", group_name, "N =", nrow(data), "\n")

+

+ if(nrow(data) < 20) {

+ return(list(ab_serial = NA, p = NA, n = nrow(data)))

+ }

+

+ model_a <- lm(z_logMeth ~ z_e3part_rev + cnsex + z_h3tage +

+ Europe_vs_Others + z_cngew + z_h3isei, data = data)

+ a_coef <- coef(model_a)["z_e3part_rev"]

+

+ model_b1 <- lm(z_son5kon ~ z_logMeth + cnsex + z_son5mon +

+ Europe_vs_Others + z_cngew + z_h3isei, data = data)

+ b1_coef <- coef(model_b1)["z_logMeth"]

+

+ model_b2 <- lm(z_son5iq ~ z_son5kon + cnsex + Europe_vs_Others + z_cngew + z_h3isei,

+ data = data)

+ b2_coef <- coef(model_b2)["z_son5kon"]

+

+ ab_serial <- a_coef * b1_coef * b2_coef

+

+ boot_serial <- function(d, indices) {

+ d_sub <- d[indices, ]

+ a_boot <- coef(lm(z_logMeth ~ z_e3part_rev + cnsex + z_h3tage +

+ Europe_vs_Others + z_cngew + z_h3isei, data = d_sub))["z_e3part_rev"]

+ b1_boot <- coef(lm(z_son5kon ~ z_logMeth + cnsex + z_son5mon +

+ Europe_vs_Others + z_cngew + z_h3isei, data = d_sub))["z_logMeth"]

+ b2_boot <- coef(lm(z_son5iq ~ z_son5kon + cnsex + Europe_vs_Others +

+ z_cngew + z_h3isei, data = d_sub))["z_son5kon"]

+ return(a_boot * b1_boot * b2_boot)

+ }

+

+ set.seed(2026)

+ boot_result <- boot::boot(data, boot_serial, R = 500)

+ boot_se <- sd(boot_result$t, na.rm = TRUE)

+ p_serial <- 2 * (1 - pnorm(abs(ab_serial / boot_se)))

+

+ return(list(ab_serial = ab_serial, p = p_serial, n = nrow(data)))

+ }

> cg_serial <- run_serial_group(data_complete[data_complete$cngrup == 0, ], "Control Group")

Control Group N = 39

> ig_serial <- run_serial_group(data_complete[data_complete$cngrup == 1, ], "Intervention Group")

Intervention Group N = 47

> cat("\n--- Results (MNAR) ---\n")

--- Results (MNAR) ---

> cat("Control Group:\n")

Control Group:

> cat(" N:", cg_serial$n, "\n")

N: 39

> cat(" Serial indirect:", round(cg_serial$ab_serial, 4), "\n")

Serial indirect: -0.0555

> cat(" p-value:", round(cg_serial$p, 4), "\n\n")

p-value: 0.384

> cat("Intervention Group:\n")

Intervention Group:

> cat(" N:", ig_serial$n, "\n")

N: 47

> cat(" Serial indirect:", round(ig_serial$ab_serial, 4), "\n")

Serial indirect: -0.018

> cat(" p-value:", round(ig_serial$p, 4), "\n\n")

p-value: 0.3736

> if(!is.na(cg_serial$ab_serial) & !is.na(ig_serial$ab_serial)) {

+ diff_serial <- ig_serial$ab_serial - cg_serial$ab_serial

+ cat("Difference (IG - CG):", round(diff_serial, 4), "\n")

+ }

Difference (IG - CG): 0.0375
