# Supplementary material for: Early DNA methylation at the NGFI-A binding site of the NR3C1 1F promoter predicts cognitive functions at age five: Evidence from the Parents as Teachers intervention in the ZEPPELIN study
Source: PLoS One. 2026 Jul 20;21(7):e0344401. doi: 10.1371/journal.pone.0344401 (PMC13384299; doi:10.1371/journal.pone.0344401)
Supplement: Attachment — Submitted filename: Response to Reviewers.docx [file pone.0344401.s003.docx]

Manuscript ID: PONE-D-26-08262

Title: Early DNA methylation at the NGFI-A binding site of the NR3C1 1F promoter predicts cognitive functions at age five: evidence from the Parents as Teachers intervention in the ZEPPELIN study

Journal: PLOS ONE

Dear Dr. Singh and Reviewers,

Thank you for the careful reading of our manuscript and for the valuable comments. We have addressed all points raised by the Academic Editor and the reviewers. Below we provide a detailed point-by-point response.

All changes are highlighted in the revised manuscript (track changes version). A clean version without tracked changes is also provided.

We hope that the reviewers and the Academic Editor will be satisfied with our revisions. We thank you again for considering our article for publication in PLOS ONE.

Kind regards,

Elena Gardini, PhD

1. Clarification of Study Sample and Relation to Prior Work

• Specify whether the current sample is a subset of the full cohort.

• Provide a CONSORT‑style flow diagram showing sample derivation.

• Compare the analytic sample to the full cohort on key demographic variables.

• Clearly delineate what is novel in this manuscript relative to Gardini et al. (2020).

• Ensure that any reused figures or materials are properly cited and, if necessary, accompanied by copyright permissions.

***Authors’ response***: we thank the reviewers for bring up these issues.

- *Specify whether the current sample is a subset of the full cohort*

The sample is a subset of the full cohort. We have specified the sample characteristics in the section “Participants”.

**Corrected section** (lns. 125, p. 7): Participants in the current study are a subset drawn from the full ZEPPELIN cohort, as only families who signed informed consent to provide saliva samples of their child were included (see Fig. 1). The full ZEPPELIN cohort includes 248 psychosocially at-risk families identified through an interdisciplinary network of family counseling and health professionals in the metropolitan area of the city of Zurich at three project sites in the canton of Zurich. Families were recruited before or shortly after childbirth based on their answers on a risk-screening questionnaire (38). Risk factors were parental risks (low level of education, early parenthood, alcohol or drug abuse, sickness and disabilities), familial risks (single parenthood, partnership conflicts), social risks (lack of social integration, dissocial environment), material risks (unemployment, financial problems, confined living space) and child related risks (high-risk pregnancy, health issues, prematurity). Families were included if they showed at least two risk factors which were not compensated by protective factors, such as support from the extended family, stable and reliable parental figures, or clear and transparent family structures. Exclusion criteria were no permanent residency permit, severe illness or disability of the child, severe illness or disability of the parent requiring inpatient and long-term psychiatric treatment, and other intensive treatments or child protection procedures. After agreeing to participate in the study, families were assigned to the IG (n=132) or CG (n=116). The assignment was performed using stratified block randomization. Strata were project site, cumulative psychosocial risk factors assessed using the short screening, Swiss nationality, family structure (single parent: yes/no), and German-language proficiency (interpreter: yes/no) (39). From the 248 families recruited, 131 agreed to provide saliva samples of their children. A total of 135 saliva samples were collected (IG = 72, CG = 63). After biochemical analyses, three samples from the CG were excluded due to insufficient sequencing coverage. Among the excluded samples, one was a twin (from a twin pair) and two were from single-child families. Consequently, the final dataset includes N = 132 saliva samples (IG = 72, CG = 60).

At recruitment, all families lived in the Canton of Zurich, Switzerland, and 79.5% primarily spoke a non-German language (39).

- *Provide a CONSORT style flow diagram showing sample derivation*

We included a CONSORT flow diagram (Fig. 1, lns. 152, p. 8).

- *Compare the analytic sample to the full cohort on key demographic variables*

We have compared the analytical sample to the full cohort for demographic variables and other key study variables (the results are reported in the Supporting Information file).

**Added section** (lns. 285, p. 14): The analytical sample of the present study (N = 132) did not differ from the full ZEPPELIN cohort (N = 248) regarding the strata used for the randomization process, nor regarding child age at recruitment, sex, and SES (see Supporting Information).

- *Clearly delineate what is novel in this manuscript relative to Gardini et al. (2020)*

We have clarified what is novel compared to Gardini et al., 2020 at the end of the introduction.

**Corrected section** (lns. 92, p. 6): Building on this concept, Gardini et al. (36) tested whether participation in a randomized controlled trial of the Parents as Teachers (PAT) psychosocial intervention (37) among families living in disadvantaged conditions was associated with reduced methylation at the NGFI-A binding domain (hereafter DNA methylation). The authors showed that PAT was associated with decreased DNA methylation, whereas parental disagreement (PD) and maternal depression symptoms were associated with increased DNA methylation and behavioral problems at age three. Furthermore, an indirect association between PD and internalizing symptoms via DNA methylation was observed.

The present study extends these findings in three key directions. First, we examine the prospective link between DNA methylation at age three and later cognitive outcomes at age five, which has not been previously investigated. Second, we test whether DNA methylation mediates the association between early stressors (parental disagreement) and cognitive performance. Third, we replicate the association between the PAT intervention and DNA methylation using an imputed dataset to handle missing data.

We report three main findings: (1) higher DNA methylation levels at age three are associated with lower cognitive performance at age five, providing preliminary empirical evidence for a prospective link between DNA methylation and cognitive outcomes in childhood; (2) DNA methylation mediates the association between parental disagreement and concentration problems; and (3) the PAT intervention is associated with lower DNA methylation levels. These findings may underscore the role of early psychosocial conditions and targeted home-visiting interventions in shaping epigenetic and cognitive development.

- *Ensure that any reused figures or materials are properly cited and, if necessary, accompanied by copyright permissions.*

We have added the reference for Figures 1 and 2. Figure 1 (CONSORT flow diagram) was modified from Schaub et al. and Figure 2 was reprinted from Gardini et al., 2020. Because we reused Figure 2, we have verified the Cambridge university press copyright permissions and we confirm that the article was distributed under the terms of the Creative Commons CC BY license and that no permission is required.

2 Missing Data and Imputation Procedures

*• Justify the choice of imputation method given the data structur.*

*• Describe assumptions regarding MCAR/MAR/MNAR*

*• Provide sensitivity analyses (e.g., complete‑case analyses) to demonstrate robustness of results.*

***Authors’ response***: We thank the reviewer for raising this concern. We agree that PCA-based imputation is not best suited for the variables of the present study. We therefore replaced it with a more appropriate method: multiple imputation (MI) under the Missing At Random (MAR) assumption. We used Predictive Mean Matching (PMM) as the imputation method for all variables, as it makes no distributional assumptions and appropriately handles both continuous and ordinal variables. A total of m = 20 imputed datasets were generated, and results were combined across imputations using Rubin's rules.

We then repeated all analyses using only the Complete Cases (CC) dataset (n=86) to assess the robustness of results. Finally, conducted a sensitivity analysis assuming a Missing Not At Random (MNAR) mechanism. We focused on parental disagreement and socioeconomic status (SES) because they had the highest proportions of missing data (parental disagreement: 16.7%; SES at T3: 12.1%) and because it is theoretically plausible that families with higher levels of parental disagreement, as well as those with lower SES, might be less likely to respond, making the missing data potentially MNAR. For parental disagreement, we applied a delta adjustment of +1 standard deviation to the imputed values, reflecting the assumption that missing values would have been systematically higher (i.e., greater disagreement). For SES, we applied a delta adjustment of –1 standard deviation to the imputed values, reflecting the assumption that families with lower SES were more likely to have missing data.

**Corrected section** (lns. 272, p. 14): To handle missing data, we employed multiple imputation (MI) under the Missing At Random (MAR) assumption using Predictive Mean Matching (PMM). A total of 20 imputed datasets were generated, and results were combined using Rubin's rules. Across all variables, 6.5% of data points were missing. Missingness was concentrated in parental disagreement (16.7%), cognitive functions (15.2%) and SES (12.1%). Other variables had minimal (<3%) or no missing data. To assess the robustness of our results, we conducted complete-case analysis (listwise deletion) and Missing Not At Random (MNAR) analyses applying a delta adjustment of +1 standard deviation to imputed values of parental disagreement (higher values indicating more disagreement) and –1 standard deviation to imputed values of socioeconomic status (lower values indicating lower SES).

As described in the Results section, the findings were robust across the three analytical approaches (multiple imputation under MAR, complete-case analysis, and MNAR sensitivity analysis), with the main effects remaining consistent in direction and significance.

**Added section** (lns. 332, p. 18): Results were largely consistent across multiple imputation (MAR) and complete-case analyses, with two exceptions. First, the effect of the intervention on methylation was significant under MAR (β = -0.375, p = 0.041) but not in complete cases (β = 0.015, p = 0.949). Second, the indirect effect of parental disagreement on concentration via methylation was significant under MAR (indirect effect = 0.064, p = 0.023) but only marginal in complete cases (indirect effect = 0.076, p = 0.076). Regarding covariates, socioeconomic status and birth weight were significantly associated with IQ in both MAR and complete-case analyses (SES: β = 0.23-0.26, p < 0.05; birth weight: β = 0.19-0.23, p < 0.05). Gender was significantly associated with concentration in both models (β = -0.44 to -0.46, p < 0.05), with girls showing fewer concentration difficulties. Child's age at randomization was consistently associated with higher methylation levels (β = 0.24-0.29, p < 0.01), indicating that later study entry (i.e., older age at randomization) was related to increased DNA methylation. However, this effect did not differ between the intervention and control groups (interaction β = 0.055, p = 0.756).

Under the MNAR assumption (imputing missing SES values one standard deviation below the observed mean and missing parental disagreement values one standard deviation above the observed mean), results remained largely unchanged. Specifically, the effects of the intervention on methylation (β = -0.367, p = 0.043), the indirect effect of methylation on IQ via concentration (indirect effect = -0.078, p = 0.032), and the indirect effect of parental disagreement on concentration via methylation (indirect effect = 0.054, p = 0.032) remained significant. The association between methylation and understanding of instructions became significant under MNAR (β = 0.183, p = 0.037), while it was marginal under MAR (p = 0.062). All other conclusions, including the absence of moderated mediation and non-significant serial mediation, were consistent across both missing data assumptions.

3. Mediation, SEM, and Interpretation of Effects

• Clarify the rationale for testing mediation in the absence of an A→C association.

• Distinguish clearly between the SEM used here and the path analysis reported in the 2020 publication.

• Remove causal or predictive language (e.g., “methylation predicts IQ”).

• Avoid interpreting group‑specific effects when interaction terms are non‑significant.

• Reframe findings as exploratory associations rather than mechanistic pathways.

• Clarify the rationale for testing mediation in the absence of an A→C association.

***Authors’ response***: We thank the reviewers for highlighting this methodological point.

*• Clarify the rationale for testing mediation in the absence of an A→C association*

Following the recommendations of Hayes (2009) and Zhao et al. (2010), we adopted an indirect-only mediation framework. In this approach, mediation is established solely by the significance of the indirect effect (a × b), without requiring a significant direct effect (A → C) as a precondition. Zhao et al. (2010) explicitly classify "indirect-only mediation" as a valid pattern in which the indirect effect is significant while the direct effect is not (i.e., full mediation). This modern perspective departs from the classic Baron and Kenny (1986) approach, which required a significant direct effect as a first step. As Hayes (2009) and Zhao et al. (2010) demonstrate, the requirement of a significant direct effect is unnecessary: the sole criterion for establishing mediation is the significance of the indirect effect, which we assessed using nonparametric bootstrapping with 1,000 resamples (Hayes, 2018).

**Revised section** (lns. 255, p. 13): Following the recommendations of Hayes (51) and Zhao et al. (52), we adopted an indirect-only mediation approach and tested indirect effects regardless of the significance of the direct effects. In this framework, mediation is established solely by the significance of the indirect effect, and a significant direct effect is not required as a precondition. Zhao et al. (52) explicitly classify 'indirect-only mediation' as a valid pattern in which the indirect effect is significant while the direct effect is not.

- *Distinguish clearly between the SEM used here and the path analysis reported in the 2020 publication.*

To maintain consistency with the analytical approach used in the 2020 publication (Gardini et al.), we employed regression-based mediation analysis using the mediation package in R, which is conceptually equivalent to the PROCESS macro in SPSS. After considering the reviewer input, we chose this approach over SEM because (1) it is more appropriate for our sample size (N=132) and (2) it handles binary treatment variables (cngrup, cnsex) correctly without treating them as continuous, which is a known limitation of SEM with categorical predictors.

**Corrected section** (lns. 251, p. 12): Regression-based mediation analyses using the mediation package in R (with 1,000 bootstrap resamples) were conducted to examine the pathways linking the intervention, parental disagreement, DNA methylation, and cognitive functions.

*• Remove causal or predictive language (e.g., “methylation predicts IQ”).*

Thank you for this suggestion. We have replaced predictive language with associative language throughout the manuscript

*• Avoid interpreting group‑specific effects when interaction terms are non‑significant.*

Thank you for highlighting this issue. We delated interpretative wording around the nonsignificant interaction terms.

*• Reframe findings as exploratory associations rather than mechanistic pathways.*

Thank you for pointing this out. The revised manuscript presents more cautious language when interpreting the associations and has reframed the study under a more exploratory lens.

4. Measurement Validity of the Mediator

*• Provide a detailed description of how concentration problems were assessed.*

*• Discuss reliability, validity, and potential situational influences.*

*• Substantially temper claims about attentional mechanisms given the limitations of this measure.*

***Authors' response***: We thank the reviewer for raising this important concern regarding the measurement of concentration problems. We have revised the Methods section to add detailed information about the assessment procedure (including examiner training, and concurrent validity with IQ scores). In addition, we have better acknowledged the limitations of this measurement in the Discussion section and tempered our claims regarding attentional mechanisms accordingly.

**Revised method section** (lns. 183, p.10): As part of the standard SON-R procedure, examiners rated the child's concentration, understanding of instructions, motivation, and cooperation on a 4-point ordinal scale ('good', 'variable', 'fair', 'poor') immediately after test administration. The test manual recommends that examiners record these qualitative observations, as they can provide valuable context for interpreting the child's performance (SON-R manual, p. 90). The examiners observed the following behaviors: (a) concentration – throughout the test, the examiner observed whether the child was attentive, easily distracted, needed to be called back to the task, and required many breaks; (b) understanding of instructions – the examiner assessed whether the child understood what to do after a non-verbal or verbal demonstration, started immediately or seemed confused, and asked for clarification; (c) motivation – the examiner noted whether the child was engaged and interested, tried to solve puzzles even when difficult, or gave up easily and lost interest; and (d) cooperation – the examiner considered whether the child followed instructions, accepted feedback ("No, not like that, look"), and tried to cooperate, or whether the child was oppositional or tried to run away.

All examiners (n = 12) were graduate students in early childhood education, applied psychology, or special education, and received specific training on the SON-R from the same instructor as part of the ZEPPELIN study. Training also included video feedback on test administration. The test was administered individually (1:1) and lasted 50 to 60 minutes.

No formal inter-rater reliability was assessed; however, the standardized training procedure ensured consistency across examiners. Following the manual's recommendation that the validity of these observational ratings can be examined through their association with test performance (SON-R manual, p. 90), we assessed concurrent validity in our sample. All four observational ratings were significantly correlated with each other (rs = 0.32–0.58, p < .01) and with children's IQ scores (rs = 0.27–0.41, p < 0.01), supporting their internal consistency and concurrent validity.

**Revised discussion section** (lns. 422, p.21): The assessment of concentration problems, understanding of instructions, motivation, and cooperation was based on examiner ratings following the SON-R test battery. While this is a standard procedure in the SON-R, these ratings have several inherent limitations. The ratings are subjective, as they rely on the examiner's judgment rather than on objective behavioral metrics. The ratings reflect a single time point (immediately after test administration), which may not capture variability in a child's attention across different contexts or over time. Situational factors such as the child's fatigue, mood, or test anxiety on the day of assessment could have influenced the ratings. Given the limitations outlined above, findings involving concentration problems should be interpreted with caution. Future studies should adopt a multi-method approach to determine whether DNA methylation may serve as a reliable biomarker for concentration problems, integrating repeated assessments of both methylation and attention, observational ratings across multiple time points, parent- or teacher-report questionnaires, and—where feasible—performance-based attention measures to better capture the stability and ecological validity of the observed associations.

5. Presentation and Reporting

Several presentation issues require revision:

*• Reformat Table 2 for clarity.*

*• Improve figure labeling (e.g., standardized coefficients, confidence intervals).*

*• Replace predictive terminology with associative language throughout.*

***Authors’ response***: Thank you for these valuable suggestions.

We reformatted tables 2 for clarity (lns. 305, p. 16).

We have deleted the moderated serial mediation figure and improved the labeling of the figures and tables in this revised version. We hope that our improvements satisfy the request, but we will be happy to make further improvements if the editors or reviewers judge it necessary.

We have carefully revised the entire manuscript to replace predictive language (e.g., "predicted," "determined," "led to") with associative terminology (e.g., "was associated with," "was related to," "showed an association with").
