# Supplementary material for: Early DNA methylation at the NGFI-A binding site of the NR3C1 1F promoter predicts cognitive functions at age five: Evidence from the Parents as Teachers intervention in the ZEPPELIN study
Source: PLoS One. 2026 Jul 20;21(7):e0344401. doi: 10.1371/journal.pone.0344401 (PMC13384299; doi:10.1371/journal.pone.0344401)
Supplement: Peer review history

# Peer review: Decision Letter 0

**DOI:** 10.1371/journal.pone.0344401.r001

**Authors:** Purnima Singh

14 Apr 2026

PONE-D-26-08262

Early DNA methylation at the NGFI-A binding site of the NR3C1 1F promoter predicts cognitive functions at age five: evidence from the Parents as Teachers intervention in the ZEPPELIN study

PLOS One

Dear Dr. Gardini,

Thank you for submitting your manuscript to PLOS ONE. After careful consideration, we feel that it has merit but does not fully meet PLOS ONE’s publication criteria as it currently stands. Therefore, we invite you to submit a revised version of the manuscript that addresses the points raised during the review process.

Please submit your revised manuscript by May 29 2026 11:59PM. If you will need more time than this to complete your revisions, please reply to this message or contact the journal office at plosone@plos.org. When you're ready to submit your revision, log on to https://www.editorialmanager.com/pone/ and select the 'Submissions Needing Revision' folder to locate your manuscript file.

Please include the following items when submitting your revised manuscript:

- A letter that responds to each point raised by the academic editor and reviewer(s). You should upload this letter as a separate file labeled 'Response to Reviewers'.
- A marked-up copy of your manuscript that highlights changes made to the original version. You should upload this as a separate file labeled 'Revised Manuscript with Track Changes'.
- An unmarked version of your revised paper without tracked changes. You should upload this as a separate file labeled 'Manuscript'.

If you would like to make changes to your financial disclosure, please include your updated statement in your cover letter. Guidelines for resubmitting your figure files are available below the reviewer comments at the end of this letter.

If applicable, we recommend that you deposit your laboratory protocols in protocols.io to enhance the reproducibility of your results. Protocols.io assigns your protocol its own identifier (DOI) so that it can be cited independently in the future. For instructions see: https://journals.plos.org/plosone/s/submission-guidelines#loc-laboratory-protocols. Additionally, PLOS ONE offers an option for publishing peer-reviewed Lab Protocol articles, which describe protocols hosted on protocols.io. Read more information on sharing protocols at https://plos.org/protocols?utm_medium=editorial-email&utm_source=authorletters&utm_campaign=protocols.

As the corresponding author, your ORCID iD is verified in the submission system and will appear in the published article. PLOS supports the use of ORCID, and we encourage all coauthors to register for an ORCID iD and use it as well. Please encourage your coauthors to verify their ORCID iD within the submission system before final acceptance, as unverified ORCID iDs will not appear in the published article. *Only*  the individual author can complete the verification step; PLOS staff *cannot*  verify ORCID iDs on behalf of authors.

We look forward to receiving your revised manuscript.

Kind regards,

Purnima Singh, PhD

Academic Editor

PLOS One

Journal Requirements:

When submitting your revision, we need you to address these additional requirements.

1. Please ensure that your manuscript meets PLOS ONE's style requirements, including those for file naming. The PLOS ONE style templates can be found at

https://journals.plos.org/plosone/s/file?id=wjVg/PLOSOne_formatting_sample_main_body.pdf and

https://journals.plos.org/plosone/s/file?id=ba62/PLOSOne_formatting_sample_title_authors_affiliations.pdf

2. We note that you have included the phrase “data not shown” in your manuscript. Unfortunately, this does not meet our data sharing requirements. PLOS does not permit references to inaccessible data. We require that authors provide all relevant data within the paper, Supporting Information files, or in an acceptable, public repository. Please add a citation to support this phrase or upload the data that corresponds with these findings to a stable repository (such as Figshare or Dryad) and provide and URLs, DOIs, or accession numbers that may be used to access these data. Or, if the data are not a core part of the research being presented in your study, we ask that you remove the phrase that refers to these data.

3. If the reviewer comments include a recommendation to cite specific previously published works, please review and evaluate these publications to determine whether they are relevant and should be cited. There is no requirement to cite these works unless the editor has indicated otherwise.

Additional Editor Comments:

The manuscript examines associations among early‑life stress, NR3C1 methylation at the NGFI‑A binding site, and later cognitive development within the ZEPPELIN randomized controlled trial. Both reviewers acknowledge the strengths of the longitudinal design and the integration of psychosocial and biological data. However, they also identify substantial and overlapping concerns regarding methodological clarity, analytic justification, measurement validity, and the interpretation of findings.

Below, I summarize the key points that require detailed, point‑by‑point responses:

1. Clarification of Study Sample and Relation to Prior Work

Both reviewers note that the manuscript does not clearly describe how the analytic sample was derived from the larger ZEPPELIN RCT. Please:

• Specify whether the current sample is a subset of the full cohort.

• Provide a CONSORT‑style flow diagram showing sample derivation.

• Compare the analytic sample to the full cohort on key demographic variables.

• Clearly delineate what is novel in this manuscript relative to Gardini et al. (2020).

• Ensure that any reused figures or materials are properly cited and, if necessary, accompanied by copyright permissions.

2. Missing Data and Imputation Procedures

Reviewer 1 raises important concerns about the use of PCA‑based imputation for ordinal variables and the absence of discussion regarding missingness mechanisms. Please:

• Justify the choice of imputation method given the data structure.

• Describe assumptions regarding MCAR/MAR/MNAR.

• Provide sensitivity analyses (e.g., complete‑case analyses) to demonstrate robustness of results.

3. Mediation, SEM, and Interpretation of Effects

Both reviewers independently highlight a central issue: the manuscript interprets an indirect effect (methylation → concentration problems → IQ) despite the absence of a direct association between methylation and IQ.

To address this:

• Clarify the rationale for testing mediation in the absence of an A→C association.

• Distinguish clearly between the SEM used here and the path analysis reported in the 2020 publication.

• Remove causal or predictive language (e.g., “methylation predicts IQ”).

• Avoid interpreting group‑specific effects when interaction terms are non‑significant.

• Reframe findings as exploratory associations rather than mechanistic pathways.

If you wish to retain the mediation model, please situate it within the methodological literature on “indirect‑only mediation” and justify its appropriateness for this context.

4. Measurement Validity of the Mediator

Reviewer 1 expresses strong concern about the psychometric adequacy of the “concentration problems” variable, which is central to your mediation model. The manuscript must:

• Provide a detailed description of how concentration problems were assessed.

• Discuss reliability, validity, and potential situational influences.

• Substantially temper claims about attentional mechanisms given the limitations of this measure.

This issue should be addressed prominently in both Methods and Discussion.

5. Presentation and Reporting

Several presentation issues require revision:

• Reformat Table 2 for clarity.

• Improve figure labeling (e.g., standardized coefficients, confidence intervals).

• Replace predictive terminology with associative language throughout.

[Note: HTML markup is below. Please do not edit.]

Reviewers' comments:

Reviewer's Responses to Questions

**Comments to the Author**

1. Is the manuscript technically sound, and do the data support the conclusions?

The manuscript must describe a technically sound piece of scientific research with data that supports the conclusions. Experiments must have been conducted rigorously, with appropriate controls, replication, and sample sizes. The conclusions must be drawn appropriately based on the data presented.

Reviewer #1: No

Reviewer #2: Yes

**********

2. Has the statistical analysis been performed appropriately and rigorously?

Reviewer #1: No

Reviewer #2: Yes

**********

3. Have the authors made all data underlying the findings in their manuscript fully available?

The PLOS Data policy requires authors to make all data underlying the findings described in their manuscript fully available without restriction, with rare exception (please refer to the Data Availability Statement in the manuscript PDF file). The data should be provided as part of the manuscript or its supporting information, or deposited to a public repository. For example, in addition to summary statistics, the data points behind means, medians and variance measures should be available. If there are restrictions on publicly sharing data—e.g. participant privacy or use of data from a third party—those must be specified.

Reviewer #1: Yes

Reviewer #2: Yes

**********

4. Is the manuscript presented in an intelligible fashion and written in standard English?

PLOS ONE does not copyedit accepted manuscripts, so the language in submitted articles must be clear, correct, and unambiguous. Any typographical or grammatical errors should be corrected at revision, so please note any specific errors here.

Reviewer #1: Yes

Reviewer #2: Yes

**********

5. Review Comments to the Author

Please use the space provided to explain your answers to the questions above. You may also include additional comments for the author, including concerns about dual publication, research ethics, or publication ethics. (Please upload your review as an attachment if it exceeds 20,000 characters)

Reviewer #1: This study is a secondary analysis of data from the Zurich Equity Prevention Project with Parents Participation and Integration (ZEPPELIN) RCT. The same corresponding author previously examined the correlations among early life stress (ELS), Parents as Teachers (PAT) intervention, NR3C1 methylation, and child behavior problems at age three (Gardini et al. 2020, Development and Psychopathology, 34:810–822). In the current study they further examined whether methylation at the NGFI-A binding domain at age three predicts cognitive functions at age five and whether the PAT intervention modifies this association.

Major critiques:

1) In the previous paper (2020) medication was performed by path analysis, and in the current manuscript it’s claimed Structural Equation Models (SEMs) was used. Not sure it’s the same path analysis approach or not. Regardless, the model is oversimplified and the conclusion is questionable. In a well-designed study, one asks whether B is a mediator between A and C only if an association between A and C is already established. Direct and indirect effects are then inferred. Otherwise, there will be excessive false claims if only associations between A & B and between B & C were observed. The current study has this issue. For example, the authors claimed “DNA methylation levels (A) indirectly predicted IQ scores (C) through concentration problems (B)”. By Table 2 we know there were correlations between A & B and between B & C, but not between A and C. The claim of there is only indirect effect can be just wrong, as there is also likelihood that there is NO effect.

Minor critiques:

2) Although we can independent variables as predictors in regression models, it is inappropriate to say they can predict the outcome unless really prediction models were constructed and tested. It’s just correlation / association.

3) This is a secondary analysis and many parts of the content were already presented somewhere else. The authors need to pay attention on how to properly present what’s known and how to use the old materials. For example, Figure 1 is exactly the same as Figure 3 in the 2020 paper. However, I don’t see it’s clearly cited. Please check whether you need copyright permission, too.

Reviewer #2: This manuscript assessed the role of DNA methylation at the NGFI-A binding site of the NR3C1 1F promoter in shaping later cognitive development, within the context of a randomized psychosocial intervention (the PAT). The longitudinal design, focus on psychosocial conditions, and availability of data are notable strengths. However, several methodological and statistical issues limit the strength of the causal and mechanistic claims. In particular, aspects of measurement validity, statistical modeling, and interpretation require further clarification.

- It was difficult to determine from the text if this study took a subset of participants from a larger parent study of the PAT. This should be clarified, and if so, then it would be helpful to compare demographics of the subset to the larger study to determine if differences exist. A consort diagram would also be helpful to see how final sample was obtained.

- More clarification is needed on handling of missing data. PCA-based imputation assumes linear structure and so may not be appropriate for ordinal variables (e.g., concentration ratings). Missingness mechanisms (MCAR, MAR, MNAR) are also not discussed. Reporting sensitivity analyses comparing results with and without imputation would be helpful.

- The authors should be careful of language that implies causal pathways (e.g., methylation “predicts” IQ through concentration), as the present analyses are secondary and randomization does not extend to methylation levels or early-life stressors. Relatedly, group differences (IG vs CG) are frequently discussed despite non-significant interaction terms. While the sample size is reasonable, it is still underpowered for SEMs, especially with multiple covariates and group comparisons. The authors should avoid interpreting group-specific effects in the absence of significant interactions, and use more exploratory language throughout.

- Important to note, DNA methylation at age three did not directly predict IQ at age five. Instead, the central findings rely on indirect effects through concentration problems assessed concurrently with IQ. A key concern is the reliance on observer-rated concentration problems as the primary mediator linking methylation and IQ. These ratings do not appear to be from a standardized or validated measure of attention or self-regulation. Additionally, these likely reflect situational factors (e.g., test anxiety, familiarity with testing environments, examiner perceptions) rather than stable attentional capacity. The entire mediation model hinges on this variable, yet its psychometric properties are insufficiently described. Although the authors acknowledge this limitation briefly, the importance of this issue warrants stronger emphasis.

Minor Comments: Table 2 should be reformatted, as it is currently difficult to view. Also, figures would benefit from clearer labeling (e.g., standardized coefficients, confidence intervals).

**********

6. PLOS authors have the option to publish the peer review history of their article (what does this mean?). If published, this will include your full peer review and any attached files.

If you choose “no”, your identity will remain anonymous but your review may still be made public.

**Do you want your identity to be public for this peer review?**  For information about this choice, including consent withdrawal, please see our Privacy Policy.

Reviewer #1: No

Reviewer #2: No

**********

[NOTE: If reviewer comments were submitted as an attachment file, they will be attached to this email and accessible via the submission site. Please log into your account, locate the manuscript record, and check for the action link "View Attachments". If this link does not appear, there are no attachment files.]

To ensure your figures meet our technical requirements, please review our figure guidelines: https://journals.plos.org/plosone/s/figures

You may also use PLOS’s free figure tool, NAAS, to help you prepare publication quality figures: https://journals.plos.org/plosone/s/figures#loc-tools-for-figure-preparation.

NAAS will assess whether your figures meet our technical requirements by comparing each figure against our figure specifications.

# Author comment: Author response to Decision Letter 1

**DOI:** 10.1371/journal.pone.0344401.r002

29 May 2026

Manuscript ID: PONE-D-26-08262

Title: Early DNA methylation at the NGFI-A binding site of the NR3C1 1F promoter predicts cognitive functions at age five: evidence from the Parents as Teachers intervention in the ZEPPELIN study

Journal: PLOS ONE

Dear Dr. Singh and Reviewers,

Thank you for the careful reading of our manuscript and for the valuable comments. We have addressed all points raised by the Academic Editor and the reviewers. Below we provide a detailed point-by-point response.

All changes are highlighted in the revised manuscript (track changes version). A clean version without tracked changes is also provided.

We hope that the reviewers and the Academic Editor will be satisfied with our revisions. We thank you again for considering our article for publication in PLOS ONE.

Kind regards,

Elena Gardini, PhD

1. Clarification of Study Sample and Relation to Prior Work

• Specify whether the current sample is a subset of the full cohort.

• Provide a CONSORT style flow diagram showing sample derivation.

• Compare the analytic sample to the full cohort on key demographic variables.

• Clearly delineate what is novel in this manuscript relative to Gardini et al. (2020).

• Ensure that any reused figures or materials are properly cited and, if necessary, accompanied by copyright permissions.

Authors’ response: we thank the reviewers for bring up these issues.

• Specify whether the current sample is a subset of the full cohort

The sample is a subset of the full cohort. We have specified the sample characteristics in the section “Participants”.

Corrected section (lns. 125, p. 7): Participants in the current study are a subset drawn from the full ZEPPELIN cohort, as only families who signed informed consent to provide saliva samples of their child were included (see Fig. 1). The full ZEPPELIN cohort includes 248 psychosocially at-risk families identified through an interdisciplinary network of family counseling and health professionals in the metropolitan area of the city of Zurich at three project sites in the canton of Zurich. Families were recruited before or shortly after childbirth based on their answers on a risk-screening questionnaire (38). Risk factors were parental risks (low level of education, early parenthood, alcohol or drug abuse, sickness and disabilities), familial risks (single parenthood, partnership conflicts), social risks (lack of social integration, dissocial environment), material risks (unemployment, financial problems, confined living space) and child related risks (high-risk pregnancy, health issues, prematurity). Families were included if they showed at least two risk factors which were not compensated by protective factors, such as support from the extended family, stable and reliable parental figures, or clear and transparent family structures. Exclusion criteria were no permanent residency permit, severe illness or disability of the child, severe illness or disability of the parent requiring inpatient and long-term psychiatric treatment, and other intensive treatments or child protection procedures. After agreeing to participate in the study, families were assigned to the IG (n=132) or CG (n=116). The assignment was performed using stratified block randomization. Strata were project site, cumulative psychosocial risk factors assessed using the short screening, Swiss nationality, family structure (single parent: yes/no), and German-language proficiency (interpreter: yes/no) (39). From the 248 families recruited, 131 agreed to provide saliva samples of their children. A total of 135 saliva samples were collected (IG = 72, CG = 63). After biochemical analyses, three samples from the CG were excluded due to insufficient sequencing coverage. Among the excluded samples, one was a twin (from a twin pair) and two were from single-child families. Consequently, the final dataset includes N = 132 saliva samples (IG = 72, CG = 60).

At recruitment, all families lived in the Canton of Zurich, Switzerland, and 79.5% primarily spoke a non-German language (39).

• Provide a CONSORT style flow diagram showing sample derivation

We included a CONSORT flow diagram (Fig. 1, lns. 152, p. 8).

• Compare the analytic sample to the full cohort on key demographic variables

We have compared the analytical sample to the full cohort for demographic variables and other key study variables (the results are reported in the Supporting Information file).

Added section (lns. 285, p. 14): The analytical sample of the present study (N = 132) did not differ from the full ZEPPELIN cohort (N = 248) regarding the strata used for the randomization process, nor regarding child age at recruitment, sex, and SES (see Supporting Information).

• Clearly delineate what is novel in this manuscript relative to Gardini et al. (2020)

We have clarified what is novel compared to Gardini et al., 2020 at the end of the introduction.

Corrected section (lns. 92, p. 6): Building on this concept, Gardini et al. (36) tested whether participation in a randomized controlled trial of the Parents as Teachers (PAT) psychosocial intervention (37) among families living in disadvantaged conditions was associated with reduced methylation at the NGFI-A binding domain (hereafter DNA methylation). The authors showed that PAT was associated with decreased DNA methylation, whereas parental disagreement (PD) and maternal depression symptoms were associated with increased DNA methylation and behavioral problems at age three. Furthermore, an indirect association between PD and internalizing symptoms via DNA methylation was observed.

The present study extends these findings in three key directions. First, we examine the prospective link between DNA methylation at age three and later cognitive outcomes at age five, which has not been previously investigated. Second, we test whether DNA methylation mediates the association between early stressors (parental disagreement) and cognitive performance. Third, we replicate the association between the PAT intervention and DNA methylation using an imputed dataset to handle missing data.

We report three main findings: (1) higher DNA methylation levels at age three are associated with lower cognitive performance at age five, providing preliminary empirical evidence for a prospective link between DNA methylation and cognitive outcomes in childhood; (2) DNA methylation mediates the association between parental disagreement and concentration problems; and (3) the PAT intervention is associated with lower DNA methylation levels. These findings may underscore the role of early psychosocial conditions and targeted home-visiting interventions in shaping epigenetic and cognitive development.

• Ensure that any reused figures or materials are properly cited and, if necessary, accompanied by copyright permissions.

We have added the reference for Figures 1 and 2. Figure 1 (CONSORT flow diagram) was modified from Schaub et al. and Figure 2 was reprinted from Gardini et al., 2020. Because we reused Figure 2, we have verified the Cambridge university press copyright permissions and we confirm that the article was distributed under the terms of the Creative Commons CC BY license and that no permission is required.

2 Missing Data and Imputation Procedures

• Justify the choice of imputation method given the data structur.

• Describe assumptions regarding MCAR/MAR/MNAR

• Provide sensitivity analyses (e.g., complete case analyses) to demonstrate robustness of results.

Authors’ response: We thank the reviewer for raising this concern. We agree that PCA-based imputation is not best suited for the variables of the present study. We therefore replaced it with a more appropriate method: multiple imputation (MI) under the Missing At Random (MAR) assumption. We used Predictive Mean Matching (PMM) as the imputation method for all variables, as it makes no distributional assumptions and appropriately handles both continuous and ordinal variables. A total of m = 20 imputed datasets were generated, and results were combined across imputations using Rubin's rules.

We then repeated all analyses using only the Complete Cases (CC) dataset (n=86) to assess the robustness of results. Finally, conducted a sensitivity analysis assuming a Missing Not At Random (MNAR) mechanism. We focused on parental disagreement and socioeconomic status (SES) because they had the highest proportions of missing data (parental disagreement: 16.7%; SES at T3: 12.1%) and because it is theoretically plausible that families with higher levels of parental disagreement, as well as those with lower SES, might be less likely to respond, making the missing data potentially MNAR. For parental disagreement, we applied a delta adjustment of +1 standard deviation to the imputed values, reflecting the assumption that missing values would have been systematically higher (i.e., greater disagreement). For SES, we applied a delta adjustment of –1 standard deviation to the imputed values, reflecting the assumption that families with lower SES were more likely to have missing data.

Corrected section (lns. 272, p. 14): To handle missing data, we employed multiple imputation (MI) under the Missing At Random (MAR) assumption using Predictive Mean Matching (PMM). A total of 20 imputed datasets were generated, and results were combined using Rubin's rules. Across all variables, 6.5% of data points were missing. Missingness was concentrated in parental disagreement (16.7%), cognitive functions (15.2%) and SES (12.1%). Other variables had minimal (<3%) or no missing data. To assess the robustness of our results, we conducted complete-case analysis (listwise deletion) and Missing Not At Random (MNAR) analyses applying a delta adjustment of +1 standard deviation to imputed values of parental disagreement (higher values indicating more disagreement) and –1 standard deviation to imputed values of socioeconomic status (lower values indicating lower SES).

As described in the Results section, the findings were robust across the three analytical approaches (multiple imputation under MAR, complete-case analysis, and MNAR sensitivity analysis), with the main effects remaining consistent in direction and significance.

Added section (lns. 332, p. 18): Results were largely consistent across multiple imputation (MAR) and complete-case analyses, with two exceptions. First, the effect of the intervention on methylation was significant under MAR (β = -0.375, p = 0.041) but not in complete cases (β = 0.015, p = 0.949). Second, the indirect effect of parental disagreement on concentration via methylation was significant under MAR (indirect effect = 0.064, p = 0.023) but only marginal in complete cases (indirect effect = 0.076, p = 0.076). Regarding covariates, socioeconomic status and birth weight were significantly associated with IQ in both MAR and complete-case analyses (SES: β = 0.23-0.26, p < 0.05; birth weight: β = 0.19-0.23, p < 0.05). Gender was significantly associated with concentration in both models (β = -0.44 to -0.46, p < 0.05), with girls showing fewer concentration difficulties. Child's age at randomization was consistently associated with higher methylation levels (β = 0.24-0.29, p < 0.01), indicating that later study entry (i.e., older age at randomization) was related to increased DNA methylation. However, this effect did not differ between the intervention and control groups (interaction β = 0.055, p = 0.756).

Under the MNAR assumption (imputing missing SES values one standard deviation below the observed mean and missing parental disagreement values one standard deviation above the observed mean), results remained largely unchanged. Specifically, the effects of the intervention on methylation (β = -0.367, p = 0.043), the indirect effect of methylation on IQ via concentration (indirect effect = -0.078, p = 0.032), and the indirect effect of parental disagreement on concentration via methylation (indirect effect = 0.054, p = 0.032) remained significant. The association between methylation and understanding of instructions became significant under MNAR (β = 0.183, p = 0.037), while it was marginal under MAR (p = 0.062). All other conclusions, including the absence of moderated mediation and non-significant serial mediation, were consistent across both missing data assumptions.

3. Mediation, SEM, and Interpretation of Effects

• Clarify the rationale for testing mediation in the absence of an A→C association.

• Distinguish clearly between the SEM used here and the path analysis reported in the 2020 publication.

• Remove causal or predictive language (e.g., “methylation predicts IQ”).

• Avoid interpreting group‑specific effects when interaction terms are non‑significant.

• Reframe findings as exploratory associations rather than mechanistic pathways.

• Clarify the rationale for testing mediation in the absence of an A→C association.

Authors’ response: We thank the reviewers for highlighting this methodological point.

• Clarify the rationale for testing mediation in the absence of an A→C association

Following the recommendations of Hayes (2009) and Zhao et al. (2010), we adopted an indirect-only mediation framework. In this approach, mediation is established solely by the significance of the indirect effect (a × b), without requiring a significant direct effect (A → C) as a precondition. Zhao et al. (2010) explicitly classify "indirect-only mediation" as a valid pattern in which the indirect effect is significant while the direct effect is not (i.e., full mediation). This modern perspective departs from the classic Baron and Kenny (1986) approach, which required a significant direct effect as a first step. As Hayes (2009) and Zhao et al. (2010) demonstrate, the requirement of a significant direct effect is unnecessary: the sole criterion for establishing mediation is the significance of the indirect effect, which we assessed using nonparametric bootstrapping with 1,000 resamples (Hayes, 2018).

Revised section (lns. 255, p. 13): Following the recommendations of Hayes (51) and Zhao et al. (52), we adopted an indirect-only mediation approach and tested indirect effects regardless of the significance of the direct effects. In this framework, mediation is established solely by the significance of the indirect effect, and a significant direct effect is not required as a precondition. Zhao et al. (52) explicitly classify 'indirect-only mediation' as a valid pattern in which the indirect effect is significant while the direct effect is not.

• Distinguish clearly between the SEM used here and the path analysis reported in the 2020 publication.

To maintain consistency with the analytical approach used in the 2020 publication (Gardini et al.), we employed regression-based mediation analysis using the mediation package in R, which is conceptually equivalent to the PROCESS macro in SPSS. After considering the reviewer input, we chose this approach over SEM because (1) it is more appropriate for our sample size (N=132) and (2) it handles binary treatment variables (cngrup, cnsex) correctly without treating them as continuous, which is a known limitation of SEM with categorical predictors.

Corrected section (lns. 251, p. 12): Regression-based mediation analyses using the mediation package in R (with 1,000 bootstrap resamples) were conducted to examine the pathways linking the intervention, parental disagreement, DNA methylation, and cognitive functions.

• Remove causal or predictive language (e.g., “methylation predicts IQ”).

Thank you for this suggestion. We have replaced predictive language with associative language throughout the manuscript

• Avoid interpreting group specific effects when interaction terms are non significant.

Thank you for highlighting this issue. We delated interpretative wording around the nonsignificant interaction terms.

• Reframe findings as exploratory associations rather than mechanistic pathways.

Thank you for pointing this out. The revised manuscript presents more cautious language when interpreting the associations and has reframed the study under a more exploratory lens.

4. Measurement Validity of the Mediator

• Provide a detailed description of how concentration problems were assessed.

• Discuss reliability, validity, and potential situational influences.

Attachment Submitted filename: Response to Reviewers.docx

# Peer review: Decision Letter 1

**DOI:** 10.1371/journal.pone.0344401.r003

**Authors:** Purnima Singh

6 Jul 2026

<p>Early DNA methylation at the NGFI-A binding site of the NR3C1 1F promoter predicts cognitive functions at age five: evidence from the Parents as Teachers intervention in the ZEPPELIN study

PONE-D-26-08262R1

Dear Dr. Gardini,

We’re pleased to inform you that your manuscript has been judged scientifically suitable for publication and will be formally accepted for publication once it meets all outstanding technical requirements.

Within one week, you’ll receive an e-mail detailing the required amendments. When these have been addressed, you’ll receive a formal acceptance letter and your manuscript will be scheduled for publication.

An invoice will be generated when your article is formally accepted. Please note, if your institution has a publishing partnership with PLOS and your article meets the relevant criteria, all or part of your publication costs will be covered. Please make sure your user information is up-to-date by logging into Editorial Manager at Editorial Manager® and clicking the ‘Update My Information' link at the top of the page. For questions related to billing, please contact billing support.

If your institution or institutions have a press office, please notify them about your upcoming paper to help maximize its impact. If they’ll be preparing press materials, please inform our press team as soon as possible -- no later than 48 hours after receiving the formal acceptance. Your manuscript will remain under strict press embargo until 2 pm Eastern Time on the date of publication. For more information, please contact onepress@plos.org.

Kind regards,

Purnima Singh, PhD

Academic Editor

PLOS One

Additional Editor Comments (optional):

Reviewers' comments:

Reviewer's Responses to Questions

**Comments to the Author**

1. If the authors have adequately addressed your comments raised in a previous round of review and you feel that this manuscript is now acceptable for publication, you may indicate that here to bypass the “Comments to the Author” section, enter your conflict of interest statement in the “Confidential to Editor” section, and submit your "Accept" recommendation.

Reviewer #1: All comments have been addressed

**********

2. Is the manuscript technically sound, and do the data support the conclusions?

The manuscript must describe a technically sound piece of scientific research with data that supports the conclusions. Experiments must have been conducted rigorously, with appropriate controls, replication, and sample sizes. The conclusions must be drawn appropriately based on the data presented.

Reviewer #1: Partly

**********

3. Has the statistical analysis been performed appropriately and rigorously?

Reviewer #1: Yes

**********

4. Have the authors made all data underlying the findings in their manuscript fully available?

The PLOS Data policy requires authors to make all data underlying the findings described in their manuscript fully available without restriction, with rare exception (please refer to the Data Availability Statement in the manuscript PDF file). The data should be provided as part of the manuscript or its supporting information, or deposited to a public repository. For example, in addition to summary statistics, the data points behind means, medians and variance measures should be available. If there are restrictions on publicly sharing data—e.g. participant privacy or use of data from a third party—those must be specified.

Reviewer #1: Yes

**********

5. Is the manuscript presented in an intelligible fashion and written in standard English?

PLOS ONE does not copyedit accepted manuscripts, so the language in submitted articles must be clear, correct, and unambiguous. Any typographical or grammatical errors should be corrected at revision, so please note any specific errors here.

Reviewer #1: Yes

**********

6. Review Comments to the Author

Please use the space provided to explain your answers to the questions above. You may also include additional comments for the author, including concerns about dual publication, research ethics, or publication ethics. (Please upload your review as an attachment if it exceeds 20,000 characters)

Reviewer #1: My comments were addressed.

My comments were addressed.

My comments were addressed.

My comments were addressed.

**********

7. PLOS authors have the option to publish the peer review history of their article (what does this mean?). If published, this will include your full peer review and any attached files.

If you choose “no”, your identity will remain anonymous but your review may still be made public.

**Do you want your identity to be public for this peer review?**  For information about this choice, including consent withdrawal, please see our Privacy Policy.

Reviewer #1: No

**********

# Editor report: Acceptance letter

**DOI:** 10.1371/journal.pone.0344401.r004

**Authors:** Purnima Singh

PONE-D-26-08262R1

PLOS One

Dear Dr. Gardini,

I'm pleased to inform you that your manuscript has been deemed suitable for publication in PLOS One. Congratulations! Your manuscript is now being handed over to our production team.

At this stage, our production department will prepare your paper for publication. This includes ensuring the following:

* All references, tables, and figures are properly cited

* All relevant supporting information is included in the manuscript submission,

* There are no issues that prevent the paper from being properly typeset

You will receive further instructions from the production team, including instructions on how to review your proof when it is ready. Please keep in mind that we are working through a large volume of accepted articles, so please give us a few days to review your paper and let you know the next and final steps.

Lastly, if your institution or institutions have a press office, please let them know about your upcoming paper now to help maximize its impact. If they'll be preparing press materials, please inform our press team within the next 48 hours. Your manuscript will remain under strict press embargo until 2 pm Eastern Time on the date of publication. For more information, please contact onepress@plos.org.

You will receive an invoice from PLOS for your publication fee after your manuscript has reached the completed accept phase. If you receive an email requesting payment before acceptance or for any other service, this may be a phishing scheme. Learn how to identify phishing emails and protect your accounts at https://explore.plos.org/phishing.

If we can help with anything else, please email us at customercare@plos.org.

Thank you for submitting your work to PLOS ONE and supporting open access.

Kind regards,

PLOS ONE Editorial Office Staff

on behalf of

Dr. Purnima Singh

Academic Editor

PLOS One
